# Study protocol for a mixed-methods pilot of a physiotherapy plus education program for inpatients with major depressive disorder: Feasibility and preliminary effects

**DOI:** 10.1371/journal.pone.0326012

**Published:** 2025-11-06

**Authors:** José Lesmes Poveda-López, Carolina Jiménez-Sánchez, Raquel Lafuente-Ureta, Bárbara Marco-Gómez, Ana Villagrasa-Cantín, Sara Pérez-Mansilla, Marta Guarch-Rubio, Juan Francisco Roy

**Affiliations:** 1 Department of Physical Therapy, Faculty of Health Sciences, Universidad San Jorge, Villanueva de Gállego, Zaragoza, Spain; 2 Department of Psychiatry, Royo Villanova Hospital, Health Service of Aragón, Universidad de Zaragoza, Zaragoza, Spain; 3 Department of Psychology, Faculty of Health Sciences, Universidad San Jorge, Villanueva de Gállego, Zaragoza, Spain; 4 Faculty of Health Sciences, Universidad Internacional de La Rioja, Spain; Federal University of Ceara, BRAZIL

## Abstract

**Background:**

Major depressive disorder is a mood disorder with significant psychological and physical symptoms that can lead to disability and other severe consequences. This disorder is influenced by both genetic and environmental factors, causing neurotransmitter imbalances and inflammation. Given mayor depressive disorder’s high prevalence and impact, it is crucial to implement a health promotion and intervention program aimed at this disorder. Investigating the feasibility of physical therapy, including therapeutic exercise and health education, compared to psychiatric and psychological approaches is an essential component of this program and of improving the quality of life for patients affected by mayor depressive disorder.

**Methods:**

A concurrent nested mixed-methods study with quantitative dominance will be conducted. The quantitative study will be a quasi-experimental pilot study with a pre-post design. This study will additionally include a qualitative narrative design. Initial and post-intervention evaluations will include sociodemographic and clinical data. Quantitative data will be collected using the EQ-5D-3L, MADRS, NRS, GSE, and GCPC-UN-ESU questionnaires. These tools assess health status, depression severity, pain intensity, self-efficacy, and satisfaction levels. Qualitative data will be collected from focus groups with 6–8 participants. The question guide for patients will cover their experiences with their illness and intervention, while the guide for professionals will cover their perceptions of patient management and observed barriers and facilitators. All participants will receive the same evidence-based intervention over 3–6 weeks, with 2 weekly sessions of approximately 45 minutes each. Each session will consist of therapeutic exercise and health education to improve patients’ physical condition and self-management skills.

**Discussion:**

This study aims to evaluate the feasibility and acceptability of a physiotherapy intervention program for MDD patients. It will also provide preliminary insights into the effects on quality of life, pain, and self-efficacy, which will inform the design of a future definitive trial. The findings from this research will provide valuable scientific insights and a basis for guiding health-care policymakers on the potential inclusion of physical therapy in clinical practice guidelines and standard hospital treatments for major depressive disorders.

**Clinical Trial registration:**

NCT06983405.

## Introduction

The World Health Organization (WHO) [1] indicates that mental health is the facet of health that enables individuals to cope with stress through their abilities and skills and that it is a fundamental human right and a necessary element for community development. Declines in mental health lead to mental illness, which is characterized by distress, functional disability, an increased difficulty in basic and instrumental activities of daily living, a sedentary lifestyle, muscle atrophy, poor quality of life, low muscle strength and fatigue, pain, changes in muscle tone, cognitive and affective deterioration, social isolation, stigmatization, work absenteeism, risk of self-harm, and premature death. Beyond the affected individual, mental illness generates an increased burden on health services, resulting in higher costs and stressed operations [2].

According to the International Classification of Diseases, Tenth Revision (ICD-10) [3], and the Diagnostic and Statistical Manual of Mental Disorders, Fifth Edition (DSM-5), by the American Psychiatric Association [4], major depressive disorder (MDD) is a mood disorder characterized by profound sadness and a sustained loss of interest in any activity for at least 2 weeks. Mayor depressive disorder is associated with a significant loss of quality of life, accompanied by psychological symptoms (anhedonia, anergia, sleep, and appetite disturbances) and physical symptoms due to functional impairment. Notably, it is the most common cause of disability worldwide [5], affecting women [6] and individuals with lower economic income [7,8] more severely. In addition, it causes severe suffering and disruption to work, family, and social activities, with the most severe consequence being suicide [9,10]. MDD’s clinical manifestation may include a single or recurrent episodes over more than two years, ranging from mild-moderate to severe [11]. Each of these issues has a significant impact on the population and requires a comprehensive approach to its management [12].

Critically, MDD is a multifactorial disease that is influenced by genetic and environmental factors [13]. The presence of these factors leads to a decrease in the neurotransmitters, namely serotonin, norepinephrine, and dopamine. This dysfunction is mediated by hyperactivity of the hypothalamic-pituitary-adrenal (HPA) axis, which is responsible for stress control and the cortisol regulation, and a decrease in the morphological volume of the hippocampus due to reduced dendrites and neuronal networks [14,15]. Furthermore, MDD is considered an inflammatory disease due to the increased levels of pro-inflammatory cytokines in the blood of these patients caused by the activation of the HPA axis [5,16].

The prevalence of mental health disorders is 17% [17] in Europe, reaching 27.4% in Spain [1]. MDD is the mental illness with the highest incidence rate in the adult population, affecting 6.4% of the European population and 4.1% of the Spanish population [18]. Notably, in the first year after the COVID-19 pandemic, the prevalence of mental illnesses, especially depressive and anxiety disorders, increased by 25% worldwide [19].

The aging process of the population must also be considered. Currently, 19% of the population in Spain is over 65 years old, with this proportion expected to increase to more than 25% over the next 10 year. In this population, poor mental health status leads to a higher risk of comorbidity and disability [20], loneliness, stigmatization, and institutionalization [21,22], all of which contribute to an increased risk of mortality [23,24]. An aging population thus requires closer monitoring due to the severity of the associated repercussions and the increase in health-care costs and resource consumption [25].

The usual treatments for MDD are multimodal and include primary and specialized medical and psychological care [26–28], usually combined with pharmacological [29–31] treatment and, to a lesser extent, physical therapy [32,33]. Although pharmacology can be effective, lack of adherence and adverse effects can compromise the treatment’s effectiveness and the patient’s autonomy [34]. Psychotherapy, including techniques such as cognitive-behavioral therapy, is a common approach for treating the affective and emotional symptoms of MDD [35,36]. Physical therapists, meanwhile, deal with movement problems related to age, injury, pain, or illness, addressing myriad physical, psychological, emotional, and social aspects in order to prevent, maintain, and restore movement and functional capacity throughout a patient’s life. Within a mental health context, physical therapy must focus on the needs of the population and work in multimodal teams to improve patients’ well-being while always adhering to ethical principles [37]. Thus, physical therapy can enhance the body-mind connection through health education [38] and movement therapy, increasing patients’ awareness of their capabilities and needs [39]. In so doing, it addresses physical symptoms such as pain, strengthens the patient [40–42], and improves their mood and functionality through the release of endorphins [43]. In sum, therapeutic exercise ensures professional and coordinated treatment that enhances the patient’s emotional and physical condition [33].

### Justification

Given the high prevalence and severe impact of MDD on adult patients’ health, the community, and the health-care system, it is crucial to implement a multimodal coordinated program of health promotion, intervention, and education. The WHO emphasizes the importance of mental health and recommends action plans that prioritize this facet of health by taking into account physical, social, and economic conditions and strengthening community support networks. Therefore, it is appropriate to investigate the feasibility of physical therapy treatments, including health education and therapeutic exercise, compared to exclusively psychiatric and/or psychological approaches in improving the quality of life and health status of patients with MDD in short-stay psychiatric units.

Our study aims to 1) demonstrate the feasibility and acceptability of a physical therapy intervention program, based on health education and therapeutic exercise, for adults with MDD in a short-stay psychiatric unit, 2) provide preliminary estimates of the program’s effects on patients’ quality of life, pain, and self-efficacy, and 3) explore the perceptions of disease management, barriers, and facilitators related to the multimodal treatment among the study participants and the health-care professionals of the psychiatric team.

## Materials and methods

### Design

A concurrent nested mixed-methods study with quantitative dominance will be conducted. The quantitative study will be a quasi-experimental pilot study with a pre-post design. This study will additionally include a qualitative narrative design in which focus groups will be used to analyze, through content analysis, patients’ experience of the intervention, as well as the perceptions regarding the management of their process and the barriers and facilitators encountered by the participants during the implementation of the intervention program. Furthermore, the study will investigate the perception of the professionals (both health-care and non-health-care) from the Psychiatry Department at Royo Villanova Hospital in Zaragoza regarding the proposed intervention program and how they perceive it has influenced the disease management, barriers, and facilitators for the patients admitted to their unit.

**Fig 1:**
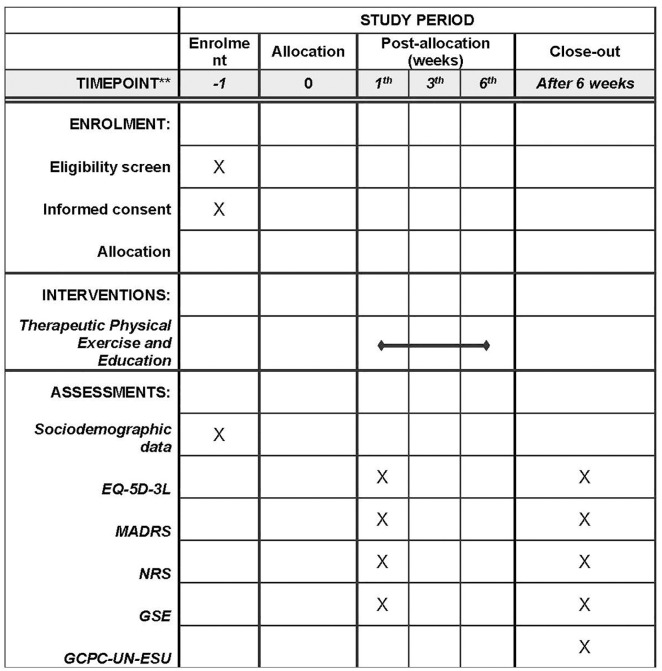
Schedule of enrollment, interventions, and assessments.

This study will be divided into two phases. Phase 1 will focus on the development of the quantitative design, and phase 2 will include the development of the qualitative design through focus groups. A schedule of enrollment, interventions, and assessments is outlined in Fig 1.

### Ethical approval and considerations

The study protocol was approved by the San Jorge University Ethic Committee (Nº 38/3/24–25) and Aragon Ethics Committee (PI25–233).

All registrations and focus group discussions will take place in private locations. Names will not be associated with notes or other study materials. Free and informed consent will be obtained from participants. Data will be documented electronically on data collection tablets. A separately completed declaration of consent will be required for all recordings made during the interviews. As part of the informed consent procedure, participants will be informed that the anonymized data may be used for publication.

To protect confidentiality, all qualitative data will be anonymized before any analyses are conducted. Each participant will be assigned a code comprising a prefix that identifies their role (e.g., patient or worker) and a consecutive number. The correspondence between codes and identities will be stored securely in a password-protected file, accessible only to the researcher responsible for coding. Importantly, this researcher will not take part in the subsequent qualitative analysis, ensuring that the analysis team works solely with anonymized transcripts. This procedure safeguards participant privacy and aligns with established ethical standards for qualitative research.

The study will be conducted in accordance with the Declaration of Helsinki and following the guidelines of the Mixed Method Reporting in Rehabilitation & Health Sciences (MMR-RHS) [44] (S1 Appendix), the Consolidated Criteria for Reporting Qualitative Research (COREQ) checklist [45] (S2 Appendix), the Transparent Reporting of Evaluations with Non-Randomized Designs (TREND) checklist [46] (S3 Appendix), the Standard Protocol Items: Recommendations for Interventional Trials (SPIRIT) [47] (S4 Appendix), and the Template for Intervention Description and Replication (TIDieR) [48] (S5 Appendix).

### Participants and eligibility criteria

Two different groups will be involved in the project: patients admitted to the short-stay unit of the Psychiatry Department at Royo Villanova Hospital, who will participate in both phases of the study (quantitative and qualitative); and the professionals from this department, who will only participate in phase 2 (qualitative).

Thus, the sample will consist of consecutive voluntary patients admitted to the short-stay unit of the Psychiatry Department at Royo Villanova Hospital who meet the following inclusion criteria: over 18 years of age; diagnosed by a physician with MDD; under regular medical, psychological, and pharmacological treatment for their illness; and no need for supervision and control by professional staff of the psychiatric unit during data collection and intervention. Meanwhile, patients will be excluded based on the following criteria: presenting with comorbid physical or mental illness whose clinical characteristics and/or severity prevent understanding and/or following physical therapy interventions, the presence of physical or mental dysfunction or disability that constitutes a total or partial contraindication for physical therapy techniques, legal incapacity, or pregnancy. Finally, dropout criteria will be considered if any subjects express the desire to withdraw from the study, if less than 80% of the physical therapy intervention sessions will be attended, if an injury prevents the continuation of the sessions, or if a new disease occurs whose diagnosis and/or severity prevents the continuation of the study or results in death.

Phase 2 will incorporate an additional sample of eight individuals who will participate in a focus group for the qualitative evaluation of professionals comprising the psychiatry professional team, including both health-care and non-health-care staff, at Royo Villanova Hospital. Participants for the focus group will be recruited through purposive sampling. Participation will be voluntary, and individuals must meet the inclusion criterion of being health-care or non-health-care professionals from the short-stay psychiatry unit at Royo Villanova Hospital. Exclusion criteria will include professionals from the unit who were not actively employed under a single, permanent, or temporary employment contract throughout the duration of the physical therapy intervention. A participant may also withdraw from the study or be excluded due to sick leave, death, or a job transfer to another unit or health-care center.

### Sample size

**Fig 2:**
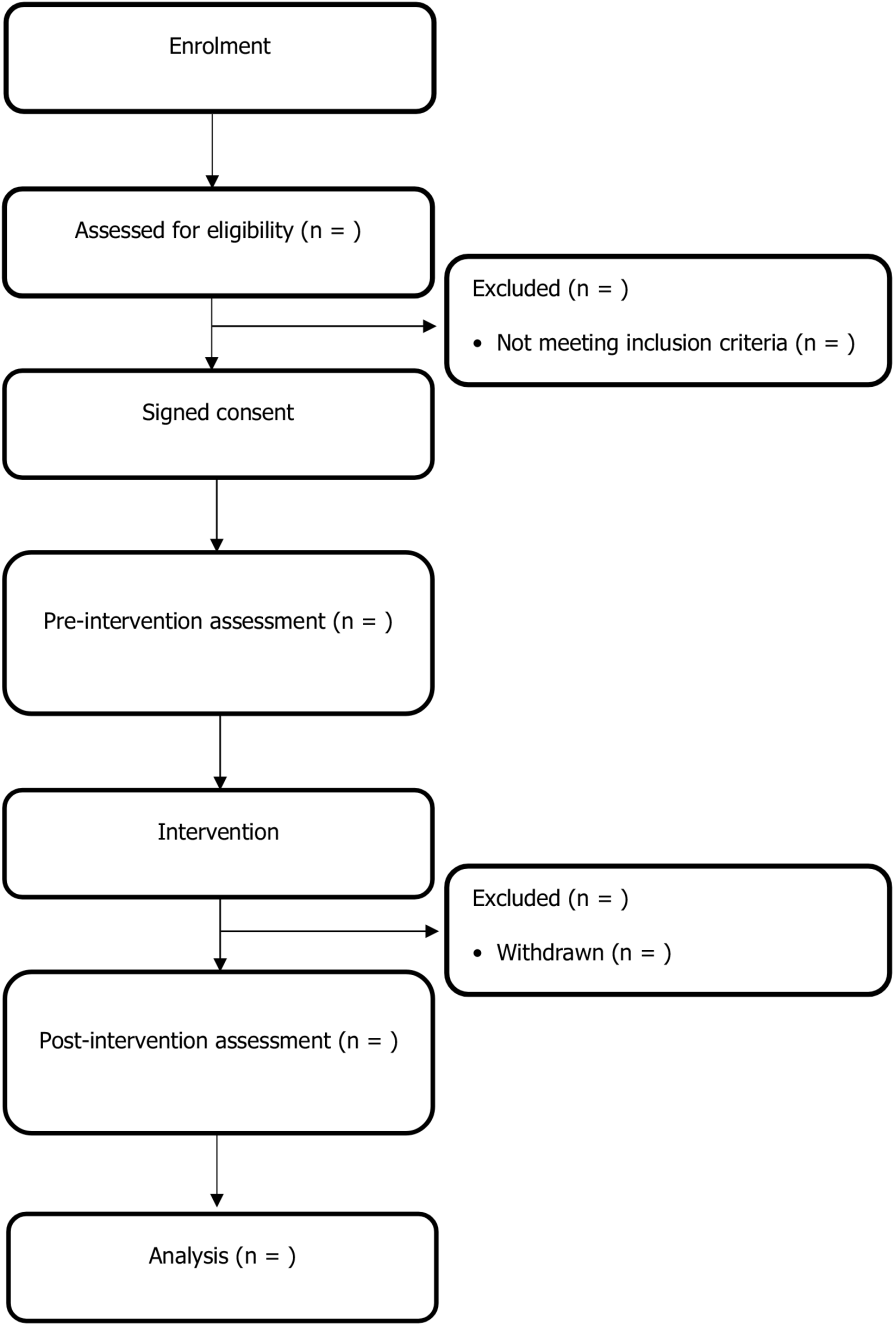
Study flowchart.

A sample size of 41 subjects will be required for phase 1 of the pilot study, as this size would ensure manageability, allow for the detection of preliminary effects, conserve resources, provide adequate variability, and be consistent with scientific precedent. This number is further considered sufficient to generate meaningful insights into the feasibility of the intervention. This study is designed as a feasibility trial with a pre-post design, using the EQ-5D-3L as the primary outcome measure. Based on previous research, a standard deviation of 0.20 and a minimally important difference (MID) of 0.10 have been assumed, resulting in a calculated effect size of Cohen’s d = 0.5 [49,50]. To detect this difference with a statistical power of 80% and a significance level of 0.05, the required sample size was estimated at 34 participants; accounting for an anticipated 20% dropout rate, the final adjusted sample size was set at 41 participants [51,52] (Fig 2). The sample size calculation was performed using Jamovi software (version 2.7.6; www.jamovi.org), specifically the power analysis module for paired-sample t-tests.

**Fig 3:**
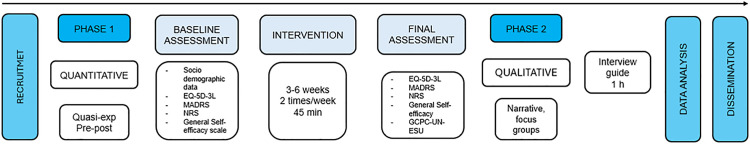
Overall project timeline and phases.

For phase 2, a purposeful sampling will be conducted to identify MMD patients and different focus groups (for patients and professionals). Each focus group will consist of 6–8 participants, and additional groups will be conducted as needed until data saturation will be reached. This approach ensures a comprehensive understanding of diverse perspectives and aligns with qualitative research standards regarding data adequacy [53,54] (Fig 3).

### Procedure

#### Feasibility and acceptability.

Feasibility and acceptability will be assessed through a combination of quantitative and qualitative indicators. Specifically, they will be evaluated by the recruitment rate, adherence to the intervention, reporting of any adverse events or protocol-related issues, and participant satisfaction. A structured feasibility logbook will be completed by the nursing staff and participants throughout the study to document attendance, dropouts, protocol deviations, and perceived barriers or facilitators. This approach will provide a comprehensive understanding of the practicality and acceptability of the intervention in a real-world setting, in line with recommendations for feasibility assessment in single-group pre-post studies [55].

#### Quantitative data collection.

An initial assessment will always be carried out before the start of the procedure as well as another at the end of the procedure before discharge from the hospital. The initial assessment will include measurements such as age, gender, occupation, and clinical data (height, weight, body mass index, medical diagnosis, pharmacological treatment, and other non-pharmacological treatments).

Quantitative data will be collected using the following questionnaires and scales. Primary outcome: the EuroQol-5D-3L Spanish version questionnaire (EQ-5D-3L) [56] Secondary outcomes: the Montgomery-Asberg Depression Rating Scale (MADRS) Spanish version [57], the numerical rating scale (NRS) [58], the General Self-Efficacy Scale (GSE) Spanish version [59], and the Health Care Satisfaction Survey for People with Chronic Illness (GCPC-UN-ESU) [60].

The EQ-5D-3L health questionnaire, which is validated for use with Spanish populations [61], can be self-administered or conducted via interview, assessing the patient’s health status across various dimensions (mobility, pain, mental health, etc.). It consists of 5 questions with 3 response options (good health = 1, some problems = 2, or health problems = 3), where responses are coded and coefficients applied to reach a reference value. Additionally, the instrument includes a 20 cm vertical visual analog scale, with values from 0 = worst health state to 100 = best health state, where the patient indicates their current perceived health status.

The MADRS is used for detecting and assessing the severity of MDD and consists of 10 questions about cognitive and affective/emotional symptoms, with response values ranging from 0 to 6 (0 = least symptomatic and 6 = most symptomatic). The rating is based on the tally of the values provided for each answer: 0–6 points no depression, 7–19 points mild depression, 20–34 points moderate depression, and 35–60 points severe depression. This tool has also been validated for use with a Spanish population [57].

The NRS for pain assessment is a tool that measures the intensity of a patient’s pain. It is a numerical scale wherein the patient is asked to rate their pain between 0 and 10. The reported values are classified as follows: no pain = 0, mild pain = 1–2, moderate pain = 3–5, severe pain = 6–8, and unbearable pain = 9–10. This tool has been validated for the assessment of pain in people with depression [62].

The GSE consists of a 10-question questionnaire that measures an individual’s perception of their ability to manage life in stressful situations, and each question is answered on a Likert scale from 1 (totally disagree) to 5 (totally agree), with scores between 27 and 38 points considered to amount to average self-efficacy. This tool has also been validated for use with a Spanish population [63].

Finally, the GCPC-UN-ESU is a validated tool for measuring satisfaction levels in Spanish-speaking individuals. It contains 19 items across 4 dimensions—care, health education, service quality, and service loyalty—and each item is scored from 1 (not satisfied) to 5 (extremely satisfied), with higher scores indicating greater satisfaction. This questionnaire will be administered anonymously and only during the post-intervention evaluation [60].

#### Qualitative data collection.

Qualitative data will be collected from two different population groups (MDD patients and professionals) using focus groups with 7–8 participants. Several focus groups will be conducted until data saturation. All focus groups will be led by one researcher, while another researcher will take field notes and log impressions from participants during the focus group. Each focus group will last approximately 1 hour, with the session being based on a question guide, and will be recorded after obtaining signed consent from each participant. Semi-structured interview guides were developed based on the validated scales and the intervention, with questions aligned with the study’s objective. The question guide in the MMD patients’ group will cover interviewees’ experiences with illness, perceptions of process management, and barriers and facilitators encountered during the intervention program. The question guide in the professionals’ groups will cover participants’ perceptions of patient disease management and the barriers and facilitators observed during the intervention program (S6 File).

To ensure methodological rigor, a multi-step validation process will be undertaken. First, content validation will be performed by a panel of experts in physiotherapy education, mental health, and qualitative research. This panel will assess whether the guide adequately reflects the study objectives and domains of interest. Second, face validation will be carried out with a small group of patients and health workers representing the target population. Specifically, they will be asked to evaluate the clarity, comprehensibility, and cultural appropriateness of the questions, as well as the overall wording and sequencing.

Following these steps, the guide will undergo a pilot test with a small sample of patients and workers with characteristics similar to the study participants. This stage will allow the researchers to assess the flow, timing, and relevance of the questions in a simulated interview setting. Cognitive interviewing techniques will be used during the piloting phase to identify potential ambiguities, unintended interpretations, or issues with response processes. Feedback from experts and participants will inform revisions, ensuring that the final version of the guide is both comprehensive and accessible.

### Intervention

This intervention is grounded in the growing body of evidence supporting the antidepressant effects of exercise [64,65] and the importance of health education in promoting self-management and long-term adherence [66].

The therapeutic exercise component will consist of 45-minute group sessions, conducted twice weekly, over a period of 3–6 weeks [67], adjusted to the participant’s length of hospital stay. Each session will include 4 main areas of focus: 1) active joint mobility, 2) strength exercises, 3) balance exercises, and 4) progressive muscle relaxation. Active joint mobility will involve gentle and controlled exercises to improve or maintain patients’ range of motion in major joints. The strength exercises will use the patients’ body weight and/or elastic bands and will involve low- to moderate-intensity exercises utilizing the participant’s own body weight and/or progressive resistance elastic bands to target major muscle groups. Notably, strength training has demonstrated moderate effects in reducing depressive symptoms [68] and may contribute to improving overall physical health in individuals with mood disorders [69]. The balance exercises will enhance patients’ stability and prevent falls, with their difficulty adjusted to each participant’s capability level. Lastly, progressive muscle relaxation will be implemented at the end of each session to reduce muscle tension and promote relaxation [70].

This exercise program will be designed to be progressive and adaptable to the individual capabilities of the patients, taking into account their clinical status and potential physical limitations. Active participation and the modification of exercises as needed will be encouraged.

The health education component will be integrated into the exercise sessions and may include brief discussions and informational materials on the following topics: 1) the relationship between physical activity and mental health, explaining potential biological and psychological mechanisms through which exercise can ameliorate depressive symptoms [71]; 2) the benefits of the specific therapeutic exercises included in the program (strength, balance, mobility) and their associated improvements around mood, physical functioning, and quality of life [72]; 3) strategies for increasing daily physical activity after hospital discharge, promoting long-term adherence [68], and the integration of exercise as a regular lifestyle component; 4) self-assessment and -monitoring techniques for physical activity and mood to foster self-efficacy and self-management [68]; and 5) information on community resources and physical activity programs available post-discharge [68].

The intervention will be delivered by professionals trained and experienced in therapeutic exercise and mental health. To ensure the fidelity of the intervention and enhance the replicability of the study, a rigorous monitoring protocol will be implemented. This protocol will entail maintaining attendance logs to record patient participation in each of the scheduled sessions. The supervising physical therapist will also use a session-specific checklist to confirm the delivery of all key components—including the therapeutic exercises, relaxation techniques, and health education modules—in each session. Any deviations or modifications to the protocol, such as a patient’s inability to perform a specific exercise due to physical discomfort or a change in their clinical status, will be meticulously documented in a dedicated log. This systematic approach will allow for a precise assessment of the intervention’s implementation, ensuring that any observed effects can be accurately attributed to the intervention and not to variations in its delivery.

Patient responses will be evaluated using depression and physical functioning rating scales, both at baseline and at the end of the intervention (S7 Table).

### Data analysis

#### Feasibility and acceptability analysis.

Feasibility and acceptability data will be recorded using Excel spreadsheets and the associated study protocol forms and documents. Most feasibility and acceptability measures will be descriptively presented using frequencies, total numbers, and percentages or proportions. The data analysis will be conducted using IBM-SPSS Statistics version 29 software (IBM Corp., Armonk, NY, USA). In addition to the descriptive analysis of the feasibility and acceptability measures, the qualitative data will be analyzed to gain deeper participant insights. NVivo software version 12 (QSR International, Melbourne, Australia) will be used to support the organization and interpretation of the qualitative data.

#### Quantitative data analysis.

The statistical analysis of the quantitative data will be performed using IBM-SPSS Statistics version 29 (IBM Corp., Armonk, NY, USA).

Data will be expressed as the mean and the standard deviation with a 95% confidence interval or as the median and an interquartile range. The Shapiro-Wilk test will be used to assess normality. For comparisons between pre- and post-intervention scores, the paired t-test will be applied if parametric assumptions are met. If these assumptions are violated, the Wilcoxon signed-rank test will be used. The significance level will be defined as p ≤ 0.05.

To address missing data, a complete case analysis (CCA) approach will be applied. This analysis will include only those participants with complete observations for both time points.

Effect size will be calculated using Cohen’s d to determine clinical significance: Insignificant, small, medium, and large differences will be reflected in effect sizes of < 0.2, 0.2–0.5, 0.5–0.8, and > 0.8, respectively [73].

Due to the limited sample size, any covariate adjustments—such as those for sex or age—will be considered exploratory and interpreted with caution.

#### Qualitative data analysis.

The qualitative data obtained from the focus groups will be analyzed using a content analysis. The recorded sessions will be fully transcribed verbatim by a single researcher. The qualitative data will be coded and analyzed using NVivo software version 12 (QSR International, Melbourne, Australia), facilitating the organization, categorization, and synthesis of information. Initially, one or more complete readings will be conducted to obtain a global understanding of the recorded information and achieve immersion in the text. Subsequently, a second word-by-word reading will be performed, during which inductive coding of the transcripts will be carried out to capture key concepts and thoughts: Meaning units will be transformed into condensed meaning units, then into codes [74]. The codes will then be grouped by their relationship and linkage into subcategories. Depending on the relationships between subcategories, the researchers may compare, combine, or organize this larger number of subcategories into a smaller number of categories and main categories [75]. This process will be conducted independently by two researchers [74].

The research team will discuss the categories and subcategories derived from the related narrations, refining them until a consensus is reached. To enhance the trustworthiness of the qualitative data, several strategies will be applied: triangulation of researchers during data analysis [76], member checking to validate interpretations with participants, maintaining a transparent audit trail of methodological decisions, and pursuing data saturation to ensure the robustness of the findings. Additionally, an integration of quantitative and qualitative data will be conducted.

### Consideration of gender perspective

Equitable inclusion across different sexes will be taken into account during recruitment to achieve a point prevalence per sex similar to the latest available data from the health sector or the general population in the city of Zaragoza or the Autonomous Community of Aragon [77]. Since MDD has a higher prevalence in the female sex [78], consideration will be given to whether there are differences according to sex in order to offer specific solutions.

Exploratory subgroup analyses will be performed, incorporating a gendered perspective as a variable of interest.

## Discussion

Despite the growing interest in physiotherapy interventions, there remains a significant gap in understanding their feasibility and acceptability among individuals with MDD, particularly during the acute phase and hospitalization. This study will significantly contribute to expanding our knowledge on how a physiotherapy intervention program based on therapeutic exercise and health education can be successfully implemented for this vulnerable population, specifically as a complement to standard psychiatric, psychological, and nursing treatment.

The findings of this research will not only provide relevant scientific evidence on the viability of the program but also valuable information to guide health policymakers in the potential implementation of physiotherapy as an integral part of the multidisciplinary approach to MDD in the hospital setting.

The marked prevalence and profound impact of MDD on the health of adult patients, in the community, and in the health system overall underscore the urgent need to explore alternative, rapid, and easily accessible treatment options [79]. Critically, depressive illness, in its progression, can intensify and/or trigger the development of physical [33] and mental [80] comorbidities, which can in turn lead to a reduced life expectancy [81].

The choice of a mixed-methods design with quantitative predominance, specifically a nested mixed-methods design, is based on the need to obtain a comprehensive understanding of the intervention’s feasibility [82]. The quantitative phase, through a pre-post pilot quasi-experimental design, will enable the evaluation of changes in quality of life, pain, self-efficacy, depression severity, and satisfaction with care. Concurrently, the qualitative phase will use focus groups with patients and health-care professionals to examine in detail their experiences with the intervention, their perceptions of disease management, and the factors that act as barriers or facilitators in the implementation of the program. This integration of quantitative and qualitative data will enable a triangulation of the findings [83], providing a richer and more contextualized understanding of the studied phenomenon, surpassing the insights offered by a unimodal approach.

It is important to emphasize that, while the existing evidence consistently suggests that moderate- to vigorous-intensity aerobic exercise has a significant positive impact on reducing depressive symptoms [33,66,79], this multimodal intervention has been carefully designed to provide an accessible and safe option for hospitalized patients in an acute phase of their illness. The program focuses on components that are likely to be well-tolerated by this population and, most importantly, lay the foundation for the adoption of more sustained physical activity after hospital discharge. The strategic combination of strength exercises [65] (which have shown to have a moderate effect in reducing depressive symptoms and can improve overall physical health), balance exercises [84] (to enhance stability and prevent falls), and progressive relaxation techniques [32] (to reduce muscle tension and promote relaxation), as well as a health education component (to foster patient autonomy, self-management skills, and a deeper understanding of their condition), comprehensively addresses multiple dimensions of physical and mental well-being.

This protocol aligns with the growing body of evidence that supports the antidepressant effects of exercise [79] and emphasizes the importance of health education in promoting self-management and long-term treatment adherence [85]. The conceptual framework is based on the understanding of the mind-body connection and how physical activity can positively influence mood through biological mechanisms, such as the release of endorphins [43], and psychological mechanisms, such as increased self-efficacy and improved bodily awareness [86,87].

It is anticipated that the implementation of this physiotherapy program will have significant practical implications. The results could provide a solid justification for the routine inclusion of physiotherapy in treatment plans for hospitalized patients with MDD. This finding could translate into a tangible preliminary insight centered on patients’ perceived quality of life; a reduction in their associated pain [88], which often coexists with depression; an increase in their self-efficacy to manage their condition; and potentially greater adherence to physical activity recommendations after discharge, which in turn could help prevent relapses [89]. On a theoretical level, this study could strengthen the evidence base for the role of physical activity and health education as effective complementary interventions in the management of MDD in an acute setting.

### Dissemination plan

The feasibility of this protocol has been carefully assessed by designing a low- to moderate-intensity intervention that can be adapted to the individual capacities of patients and the duration of their hospital stay. Various strategies—such as the verbatim transcription of the focus groups, inductive coding independently performed by two researchers, and the triangulation of data to ensure the credibility of the findings—will be employed to ensure the quality of the qualitative data.

Upon study completion and analysis finalization, the open-access repository Zenodo will house the entirety of the raw quantitative data, codebooks, and statistical analysis scripts. The findings of this study will be disseminated through several channels to maximize their impact. Primary dissemination will occur through publication in peer-reviewed scientific journals, with *PLOS ONE* being the targeted journal. Presentations at national and international conferences related to physical therapy, mental health, and health care will also be pursued. Furthermore, the results will be shared with participating health-care institutions and, if applicable, with the study participants. The potential for the development of clinical practice guidelines or recommendations based on the study’s outcomes will be explored to facilitate the integration of physical therapy into standard care for hospitalized patients with MDD.

After the publication of the findings, the study’s data will be made available upon request, with full respect for the confidentiality of all participants.

### Study amendments and termination

All amendments to the study protocol will be subject to review and approval by the Aragon Ethics Committee to ensure the continued safety and ethical integrity of the research. All approved amendments will be thoroughly documented, logging the date of approval, the nature of the changes, and the rationale for the modifications, and will be communicated to all relevant study personnel. The study may be terminated prematurely if unforeseen safety concerns arise, if participant recruitment is significantly below the targeted threshold, or if external factors impede the study’s continuation. In the event of a premature termination, all collected data will be retained and analyzed to the extent possible, and the findings will be reported in accordance with ethical guidelines and applicable regulations. The reasons for termination will be clearly documented and included in any publication of the study’s results.

### Limitations

The main expected limitation stems from the possible loss of patients during follow-up, which may require a more extensive recruitment period to reach the target sample size. Another limitation inherent in intervention studies in clinical settings is the difficulty of controlling the physical activity that patients may perform outside of structured sessions. The heterogeneity of MDD, with its various subtypes and clinical manifestations, could also introduce variability in responses to the intervention, although the specific diagnosis will be taken into account to minimize this risk. Patients’ lifestyle prior to admission (their baseline level of physical activity) could also influence the magnitude of the changes observed.

Finally, it is crucial to recognize that patients will receive standard pharmacological and psychological treatment for their condition, which will make it difficult to exclusively attribute the observed improvements to the physical therapy and health education interventions. However, the mixed-methods design will make it possible to explore the perceptions of patients and professionals about the specific contribution of physical therapy to the patients’ recovery process.

## Supporting information

S1 Appendix MMR-RHS checklist. (PDF)

S2 Appendix Coreq-checklist. (PDF)

S3 Appendix Trend-checklist. (PDF)

S4 Appendix SPIRIT-checklist. (PDF)

S5 Appendix TIDieR-checklist. (PDF)

S6 File Question guide. (PDF)

S7 Table Summary of the intervention. (PDF)

S1 File Project submitted to the ethics committee-Spanish. (DOCX)

S2 File Project submitted to the ethics committee-English. (DOCX)
