# Supplementary material for: Study protocol for a mixed-methods pilot of a physiotherapy plus education program for inpatients with major depressive disorder: Feasibility and preliminary effects
Source: PLoS One. 2025 Nov 6;20(11):e0326012. doi: 10.1371/journal.pone.0326012 (PMC12591423; doi:10.1371/journal.pone.0326012)
Supplement: S6 File — (PDF) [file pone.0326012.s006.pdf]

GUIDE OF QUESTIONS FOR THE RECIPIENTS OF THE INTERVENTION:

Generic Questions:

- Explain to us how the life process that led you to be admitted to the unit has been.
- What factors/elements have facilitated your stay in the hospital?
- What factors/elements have been a barrier during your stay in the hospital?
- What factors/elements have facilitated you in doing the physical exercise program?
- What factors/elements have been a barrier for you in doing the physical exercise program?
- Have you followed the physical exercise recommendations during the periods outside the program?
- Have you experienced any changes in your health status after the program?
- What do you think about the presence of physiotherapy in a short-stay psychiatric unit?

Self-efficacy:

- Do you consider that there have been changes in your ability to find solutions or solve problems/manage unexpected situations after the program?
- Do you consider that you achieve the things you want or meet your goals differently than you did before the start of the program?

Quality of Life:

- Do you consider that your mobility has changed after the program? Can you explain how/why?
- Do you consider that your personal care is different after the program? Can you explain how/why?
- Do you consider that the ease of doing your daily activities (work, studies, household activities, leisure time) has changed after the program? Can you explain how/why?
- Do you consider that your sensation of pain has changed after the program?
- Do you consider that your feeling of sadness has changed after the program? Can you explain how/why?
- Do you consider that your lack of interest in things, if you had it, has changed after the program? Can you explain how/why?

Satisfaction with the Program:

- What is your opinion about the way the people who carried out the activity have worked with you?

- What did you think of the place and conditions in which the activity/intervention was carried out? Could you expand your opinion on this?
- What is your opinion regarding the intervention program you received? Do you think it is appropriate/beneficial? Can you justify your answer?
- Has the program met your expectations? Would you recommend the program to other people? Why?

GUIDE OF QUESTIONS FOR PROFESSIONALS:

Generic Questions:

- What factors/elements do you consider have facilitated the patients during their stay in the hospital?
- What factors/elements have been a barrier for the patients during their stay in the hospital?
- What factors/elements have facilitated the patients in doing the physical exercise program?
- What factors/elements have been a barrier for the patients in doing the physical exercise program?
- Do you consider that they have followed the physical exercise recommendations during the periods outside the program?
- Do you consider that they have experienced any changes in their physical/psychological symptoms after the program?
- What do you think about the presence of physiotherapy in a short-stay psychiatric unit?
