# Supplementary material for: Study protocol for a mixed-methods pilot of a physiotherapy plus education program for inpatients with major depressive disorder: Feasibility and preliminary effects
Source: PLoS One. 2025 Nov 6;20(11):e0326012. doi: 10.1371/journal.pone.0326012 (PMC12591423; doi:10.1371/journal.pone.0326012)
Supplement: S7 Table — (PDF) [file pone.0326012.s007.pdf]

INTERVENTION ASPECT	DETAILED DESCRIPTION
**General Rationale**	Grounded in evidence supporting the antidepressant effects of exercise and the importance of health education in promoting self-management and long-term adherence.
**Target Population**	Participants with depressive symptomatology (intervention duration adjusted to length of hospital stay).
**Interventionists**	Professionals trained and experienced in therapeutic exercise and mental health.
**Monitoring and Evaluation**	Session adherence tracking; recording of adverse events. Response evaluation using depression and physical functioning rating scales (at baseline and at the end of the intervention).
COMPONENT: THERAPEUTIC EXERCISE	
**Specific Aim**	To alleviate depressive symptoms and improve general physical health.
**Format and Duration**	45-minute sessions, twice weekly, for a period of 3 to 6 weeks (adjustable).
**Session Content**	**1. Active joint mobility:** Gentle and controlled exercises to improve or maintain range of motion in major joints.<br>**2. Strength exercises:** Using body weight and/or elastic bands (low-to-moderate intensity). Moderate effects in reducing depressive symptoms and may contribute to improving overall physical health in individuals with mood disorders.<br>**3. Balance exercises:** To enhance stability and prevent falls (difficulty adjusted to each participant's level).<br>**4. Progressive muscle relaxation:** Implemented at the end of each session to reduce muscle tension and promote relaxation.
**Application Principles**	Progressive program, adaptable to individual capabilities, clinical status, and potential physical limitations. Encouragement of active participation and exercise modification as needed.
COMPONENT: HEALTH EDUCATION	
**Specific Aim**	To promote self-management, long-term exercise adherence, and its integration as a regular lifestyle component.
**Delivery Mode**	Integrated into exercise sessions; may include brief discussions and informational materials.
**Key Topics Covered**	**1. The relationship between physical activity and mental health:** Explaining potential biological and psychological mechanisms.<br>**2. Benefits of the specific therapeutic exercises in the program:** Concerning improvements in mood, physical functioning, and quality of life.<br>**3. Strategies for increasing daily physical activity after hospital discharge:** Promoting long-term adherence.<br>**4. Self-assessment and monitoring techniques:** For physical activity and mood to foster self-efficacy and self-management.<br>**5. Information on community resources:** And physical activity programs available post-discharge.
