# Supplementary material for: Study protocol for a mixed-methods pilot of a physiotherapy plus education program for inpatients with major depressive disorder: Feasibility and preliminary effects
Source: PLoS One. 2025 Nov 6;20(11):e0326012. doi: 10.1371/journal.pone.0326012 (PMC12591423; doi:10.1371/journal.pone.0326012)
Supplement: S1 File — (DOCX) [file pone.0326012.s008.docx]

MEMORIA DE PROYECTO DE INVESTIGACIÓN PARA EL CEICA

| **TÍTULO** | Efectividad de un programa de fisioterapia basado en ejercicio físico terapéutico y educación sanitaria dirigidos a mejorar la calidad de vida y el estado de salud en trastorno depresivo mayor: estudio de diseño mixto |
| --- | --- |
| **VERSIÓN Y FECHA** | Versión 2, mayo-2025 |
| **CENTRO DONDE SE REALIZA EL ESTUDIO** | Hospital Universitario Royo Villanova (Zaragoza) |

| **INVESTIGADOR PRINCIPAL DEL PROYECTO** | |
| --- | --- |
| **NOMBRE Y APELLIDOS*** | José Lesmes Poveda López |
| **DNI*** |  |
| **E-MAIL*** | jlpoveda@usj.es |
| **TELÉFONO*** |  |
| **PUESTO DE TRABAJO** | Personal docente e investigador |
| **SERVICIO/DEPARTAMENTO** | Grado en Fisioterapia |
| **CENTRO/ FACULTAD-UNIVERSIDAD/ OTRO** | Universidad San Jorge |

| **PROMOTOR (Imprescindible para ensayos clínicos y estudios observacionales con medicamentos)** | |
| --- | --- |
| **IDENTIFICACIÓN EMPRESA/ NOMBRE Y APELLIDOS*** |  |
| **NIF/DNI*** |  |
| **E-MAIL*** |  |
| **TELÉFONO*** |  |

| **contacto de la persona encargada de gestionar la solicitud**  *Si lo desea, añada los datos de contacto de la persona encargada de gestionar los detalles de la solicitud* | |
| --- | --- |
| **NOMBRE Y APELLIDOS** | José Lesmes Poveda López |
| **E-MAIL** | jlpoveda@usj.es |
| **TELÉFONO** |  |

Los datos de carácter personal que pudieran constar en esta comunicación serán incorporados al sistema de tratamiento del que es responsable el Instituto Aragonés de Ciencias de la Salud (IACS). Los datos serán tratados para la gestión y seguimiento de los estudios evaluados por el CEICA. Los datos serán suprimidos cuando se haya dado respuesta a la gestión y/o tramitación de la solicitud y hayan dejado de ser necesarios. Tiene derecho a acceder, rectificar y suprimir los datos, así como los demás derechos que le otorga la normativa de protección de datos ante el IACS, con domicilio en el Centro de Investigación Biomédica de Aragón. Avda. San Juan Bosco, nº 13, 500009, Zaragoza o solicitándolo a través del mail protecciondedatos.iacs@aragon.es.

**GLOSARIO**

- Anonimización: proceso por el cual deja de ser posible establecer por medios razonables el nexo entre un dato y el sujeto al que se refiere. Es aplicable también a la muestra biológica.
- BIGAN: plataforma de big data sanitario (gestionada por el IACS) que permite acceder a los datos del Sistema Aragonés de Salud de forma seudonimidaza para su uso en gestión e investigación.
- Biobanco: establecimiento público o privado, sin ánimo de lucro, que acoge una o varias colecciones de muestras biológicas de origen humano con fines de investigación biomédica, organizadas como una unidad técnica con criterios de calidad, orden y destino.
- Centro: Institución donde se realiza un estudio (hospital, centro de salud, residencia, facultad, clínica privada colegio, etc). **En caso de duda se debe hacer referencia al lugar de donde proceden los participantes** (hospital, colegio, club deportivo, etc)
- Compromiso de Confidencialidad: Documento que, de forma obligatoria, deben firmar los alumnos y residentes que desarrollan alguna actividad en el sistema público de salud (modelo establecido en la Orden SSI/81/2017).
- Consentimiento informado: manifestación de la voluntad libre y consciente válidamente emitida por una persona capaz, o por su representante autorizado, precedida de la información adecuada.
- Dictamen del CEICA: documento que acredita que el CEICA ha evaluado un proyecto de investigación y que dicho proyecto cumple con las normas legales aplicables y los criterios éticos.
- Encuesta: recogida de información en soporte físico o digital, con o sin interacción directa con el sujeto fuente.
- Entrevista: recogida de información con interacción directa con el sujeto fuente, mediante respuestas verbales.
- Equipo de investigación: Conjunto de investigadores que realizan de forma conjunta un proyecto concreto.
- Fuente primaria de datos: Cuando los datos se recogen directamente del participante en el estudio y con motivo del estudio.
- Fuente secundaria de datos: Cuando se utilizan para el estudio datos ya recogidos (y registrados, por tanto) que se obtuvieron con una finalidad diferente al estudio (asistencial, docente, etc.).
- Grupo de investigación: Conjunto de investigadores con una trayectoria común (publicaciones, financiación) dirigidos o coordinados por un Investigador Principal, agrupados en torno a una temática de investigación y no necesariamente por su vinculación asistencial o departamental.
- Hoja de información al participante: Documento por el que se informa a los potenciales participantes de la naturaleza del estudio, para que puedan otorgar su consentimiento informado.
- Intervención (estudio de intervención): cualquier actuación que se vaya a realizar sobre una persona debido a su participación en un estudio (puede ser un tratamiento farmacológico, fisioterapéutico, una intervención educativa, conductual, psicológica).
- Investigador principal: Investigador que lidera el proyecto y se hace responsable de su diseño, realización y difusión de los resultados. Si el estudio es multicéntrico debe haber un investigador principal en cada centro que se responsabilice de los pacientes, datos y/o muestras.
- Muestra biológica: cualquier material biológico de origen humano susceptible de conservación y que pueda albergar información sobre la dotación genética característica de una persona
- Práctica clínica habitual: Procedimientos que se llevan a cabo con motivos puramente asistenciales, de forma independiente de la participación o no de una persona en un estudio de investigación.
- Proyecto de investigación: procedimiento científico destinado a recabar información y formular hipótesis sobre un determinado fenómeno social o científico.
- Promotor: individuo, empresa, institución u organización responsable de iniciar, gestionar, organizar y financiar un estudio.
- Seudonimización: el tratamiento de datos personales de manera tal que ya no puedan atribuirse a un interesado sin utilizar información adicional, siempre que dicha información adicional figure por separado y esté sujeta a medidas técnicas y organizativas destinadas a garantizar que los datos personales no se atribuyan a una persona física identificada o identificable.

| 1. **ALCANCE Y FINANCIACIÓN DEL PROYECTO** | |
| --- | --- |
| ¿Es un proyecto multicéntrico? Sí  No | En caso afirmativo, se debe presentar el listado de centros completo y rellenar un compromiso del equipo investigador **por cada centro de Aragón** (ver [anexo I](#A3)) |
| ¿Dispone de financiación específica para el estudio? Sí  No | En **todos los casos** se debe rellenar el [anexo II](#A4): autorización uso de recursos. Además, en caso afirmativo, se debe presentar el presupuesto y la fuente de financiación. |

| 1. **CARACTERÍSTICAS DEL ESTUDIO** | |
| --- | --- |
| **2.1 ¿Se trata de una investigación con medicamentos**? Sí  No | |
| En caso afirmativo, escoja una opción:   1. Se trata de un estudio observacional respecto al tratamiento con medicamentos (EOM)   En este caso, especificar:  Recogida de datos prospectivos  Recogida de datos retrospectivos  Recogida transversal de datos   1. Se trata de un estudio de intervención: ensayo clínico con medicamentos   En este caso se debe presentar según instrucciones de la AEMPS (<https://www.aemps.gob.es/medicamentos-de-uso-humano/investigacionclinica_medicamentos/ensayosclinicos/#n-espanola>) | |
| **2.2 ¿Se trata de una investigación con productos sanitarios o dispositivos médicos**? Sí  No | |
| En caso afirmativo, escoja una opción:   1. Se trata de un estudio observacional respecto al uso del producto sanitario 2. Se trata de un estudio de intervención: ensayo clínico con productos sanitarios   En este caso se debe presentar según PNT del CEICA para este tipo de estudios (<https://www.iacs.es/investigacion/comite-de-etica-de-la-investigacion-de-aragon-ceica/>) | |
| **2.3 ¿Se trata de una investigación con procedimientos invasivos?**  (Definición: toda intervención realizada con fines de investigación que implique un riesgo físico o psíquico para el participante).  En caso afirmativo se debe contratar una póliza de seguros o justificar riesgo mínimo | Sí  No |
| **2.4 ¿En la investigación se incluyen menores de edad o personas incapaces de dar su consentimiento?**  En caso afirmativo se debe presentar un documento de información y consentimiento informado dirigida al tutor/representante legal/familiar y otro dirigido al menor de edad (adaptado a su capacidad). [Revisar plantilla CEICA](https://www.iacs.es/wp-content/uploads/2022/12/Doc-1-23-Anexo-Plantilla-HIP-modif-2.doc). | Sí  No |

| 1. **CARACTERÍSTICAS DEL ESTUDIO** | |
| --- | --- |
| **2.5 ¿Se utilizan muestras biológicas en el estudio?** | Sí  No |
| En caso afirmativo, escoja una o varias opciones:   1. Se utilizan muestras de excedentes asistenciales con consentimiento para el proyecto   Se debe presentar el documento de información y consentimiento ([plantilla CEICA](https://www.iacs.es/wp-content/uploads/2022/12/Doc-1-23-Anexo-Plantilla-HIP-modif-2.doc))   1. Se utilizan muestras de excedentes asistenciales sin consentimiento   Se debe justificar adecuadamente en el apartado de aspectos éticos (art. 58.2 Ley 14/2007)   1. Se recogen muestras específicamente para este estudio   Se debe presentar el documento de información y consentimiento ([plantilla CEICA](https://www.iacs.es/wp-content/uploads/2022/12/Doc-1-23-Anexo-Plantilla-HIP-modif-2.doc))   1. Se crea una colección de muestras privada   Para la creación de una nueva colección, presentar la documentación necesaria para su evaluación e indicar nº de registro (nº_____________) (ver [web CEICA](https://www.iacs.es/investigacion/comite-de-etica-de-la-investigacion-de-aragon-ceica/ceica-evaluaciones-y-otras-presentaciones/ceica-proyectos-de-investigacion/))   1. Se utilizan muestras ya recogidas en una colección de muestras privada   En este caso, identificar nº de colección y responsable:_____________________   1. Se solicitan muestras a un Biobanco autorizado. En este caso, identificar el Biobanco:_________   Se debe presentar la solicitud al biobanco | |
| **2.6 ¿Se realizan análisis genéticos?** | Sí  No |
| **2.7 ¿Se utilizan embriones, células embrionarias humanas, células o tejidos fetales humanos o bien células humanas pluripotentes obtenidas mediante reprogramación celular?**  Debe contactar con el IACS o con la institución responsable para posteriores autorizaciones | Sí  No |

| 1. **TRATAMIENTO DE DATOS PERSONALES**   Se considera **Dato personal** cualquier dato (edad, sexo) o cualquier información (numérica, alfabética, gráfica, acústica) sobre una persona física identificada o identificable; se considerará identificable toda persona cuya identidad pueda determinarse, directa o indirectamente (es decir, que no se haya anonimizado de forma irreversible en origen) | |  |
| --- | --- | --- |
| **3.1 ¿Se recogen o tratan datos de carácter personal en el estudio?** | Sí  No |  |
| En caso afirmativo, marca la opción aplicable:  Se solicita el consentimiento informado del interesado  Presentar el documento de información y consentimiento ([plantilla CEICA](https://www.iacs.es/wp-content/uploads/2022/12/Doc-1-23-Anexo-Plantilla-HIP-modif-2.doc))  Se utilizan datos obtenidos con otra finalidad que han sido seudonimizados (por ej. Historia clínica, otra investigación, otros registros) conforme a la D.A. 17 de la Ley Orgánica 3/2018.  Otros. Especificar: | |  |
| Marque las **categorías** a las que pertenecen los datos recogidos:  **datos identificativos** (Nombre, dirección, email, DNI, nº Historia clínica, teléfono, firma, IP, geolocalización, imagen/voz, otros)  **datos personales:** fecha nacimiento, lugar de nacimiento, nombre padres, lugar de trabajo, datos económicos, sexo, estado civil, hijos, titulación académica, otros.  **datos de opinión**  **datos especialmente sensibles**: salud, etnia, religión, opinión política, vida u orientación sexual, afiliación sindical, necesidades educativas especiales | |  |
| - 1. **Si los datos se recogen directamente del interesado (fuente primaria), especificar el procedimiento** (Ejemplo: entrevista, encuesta en papel, correo electrónico, teléfono, aplicaciones web, …)   Entrevista personal con el interesado y evaluación presencial por parte de personal del equipo investigador.  Se llevarán a cabo grupos focales, de forma semiestructurada, en los que se propondrán una serie de preguntas abiertas a través de las cuales se obtendrán los datos cualitativos para su posterior análisis. Dichos grupos focales se llevarán a cabo de forma presencial, y se registrarán mediante una grabadora de audio para su posterior transcripción y análisis. El uso de la grabación será única y exclusivamente para el análisis del estudio. Los registros de audio recogidos se transcribirán manualmente a un documento de Microsoft Word para poder ser leídos y analizados. Toda la información se depositará en aplicación Sharepoint de Microsoft con licencia propiedad de la Universidad San Jorge a la que sólo el equipo investigador tendrá acceso. Los documentos de Hoja de Información y Consentimiento Informado a los participantes serán entregados y recogidos por el investigador principal y en colaboración con las investigadoras colaboradoras externas Dña. Ana Villagrasa Cantín y Dña. Sara Pérez Mansilla, siendo documentos custodiados durante el estudio por el investigador principal en las instalaciones de la Universidad San Jorge.  Se seguirá el mismo procedimiento para la toma de datos personales de los profesionales participantes en sus grupos focales específicos. Esta toma de datos se hará de la fuente primaria directamente a través de entrevista previa al inicio del grupo focal.  **3.3 Si no se recogen directamente del interesado (fuente secundaria),** marque la opción y especifique:  Se recogen datos de un registro ya existente (ej. Historia Clínica) **con el consentimiento** del interesado.  Se reutilizan datos de otra investigación similar, para la cual se obtuvo el consentimiento del interesado. Se debe presentar permiso del responsable de los datos, el modelo de consentimiento con el que fueron obtenidos y el compromiso de utilización de los datos seudonimizados ([ver en la web](https://www.iacs.es/wp-content/uploads/2022/12/Doc-5.-declaracion-responsable-datos-seudonimizad.docx)).  Se utilizan datos obtenidos con otro fin y **sin consentimiento para investigación** (por ej, Historia clínica u otro registro)  En este caso, indicar:  El investigador (si es personal del centro) accede directamente a la historia clínica. Presentar permiso del responsable de los datos (si se trata de la historia clínica, se debe presentar la autorización de la dirección para el acceso a datos para este estudio)  El investigador recibe los datos ya seudonimizados Presentar el compromiso de utilización de los datos seudonimizados ([ver en la web](https://www.iacs.es/wp-content/uploads/2022/12/Doc-5.-declaracion-responsable-datos-seudonimizad.docx)).  Nota: **los investigadores que no tengan relación laboral con el hospital/centro** no tienen acceso a la historia clínica, por lo que SIEMPRE que se use esta fuente deben obtener los datos seudonimizados.  En todos los casos, explicar: origen de los datos, responsable de los mismos.  Solo la investigadora Bárbara Marco Gómez, que forma parte del centro hospitalario, tendrá acceso a la historia clínica. Los datos extraídos de la historia clínica serán la fecha de nacimiento, edad, género, profesión, talla, peso, IMC, diagnósticos médicos, tratamiento farmacológico y otros tratamientos no farmacológicos si los hubiera. Los datos serán obtenidos de la entrevista personal y evaluación específica del interesado por parte del personal investigador colaborador de la unidad de corta estancia de Psiquiatría del Hospital Universitario Royo Villanova y trasladados al cuaderno de recogida de datos (CRD) de forma seudonimizada. Los CRD serán custodiados en despacho del servicio de psiquiatría hasta entrega inmediata al investigador principal, quien los conservará siguiendo las medidas de protección adecuadas, dentro de una taquilla cerrada con llave en la Universidad San Jorge, trasladando después la información a un formato Excel en la carpeta compartida Sharepoint de Microsoft 365 de licencia de la Universidad San Jorge junto al investigador Juan Francisco Roy Delgado, pudiendo a partir de entonces ser ya consultados por el resto del equipo investigador.  Datos procedentes de BIGAN  Presentar informe de la Unidad de Biocomputación ([link a la solicitud](https://www.iacs.es/instituto-aragones-ciencias-la-salud/oficina-virtual/solicitud-de-acceso-a-datos-para-realizacion-de-un-proyecto-de-investigacion-rpi01-3a/)) y el compromiso de utilización de los datos seudonimizados ([ver en la web](https://www.iacs.es/wp-content/uploads/2022/12/Doc-5.-declaracion-responsable-datos-seudonimizad.docx)) | |  |
| **3.4 Una vez obtenida la información y los datos ¿cómo se garantiza la privacidad de los participantes?**  Sólo se utilizan datos agregados (es decir, datos que corresponden a grupos de personas y no a cada una de esas personas)  Los datos se anonimizan (los datos no pueden asociarse a una persona identificada o identificable por haberse destruido el nexo irreversiblemente con toda información que identifique al sujeto)  los datos se seudonimizan o codifican (se sustituyen los identificadores directos por un código/seudónimo que sólo conoce el equipo investigador)  Explicar cómo y quién realiza la medida adoptada:  Asignación de un código alfanumérico de identificación por parte de la investigadora Bárbara Marco Gómez. Todo documento en papel será custodiado en una taquilla bajo llave en la Universidad San Jorge por parte del investigador principal, José Lesmes Poveda López. Los datos seudonimizados estarán organizados en un libro Excel y un archivo SPSS, que contendrán una contraseña para su acceso, tanto al archivo como al ordenador. | |  |
| **3.5 Plazo de conservación de los datos:** especificar fecha de destrucción (al menos mes y año):  Julio de 2026. | |  |
| **3.6 Encargados del tratamiento (no cumplimentar en caso de datos anónimos)**  Se entiende por **encargado de tratamiento** a toda persona física o jurídica, autoridad pública, servicio u otro organismo que trate datos personales, ajeno al responsable del tratamiento (investigador).  Si un tercero (ajeno a la institución) trata datos del proyecto, será necesario firmar un **contrato de encargado de tratamiento**. Un modelo se puede descargar de <https://seguridad.salud.aragon.es/plantillas/>  Indicar qué personas van a tratar los datos recogidos, especificando quién tendrá acceso a los datos identificativos:  La investigadora Bárbara Marco Gómez será la encargada de realizar la recopilación de los datos y el tratamiento de seudonimización. Después el resto de las tareas de tratamiento de los datos ya seudonimizado será realizado por José Lesmes Poveda López y Juan Francisco Roy Delgado. El resto del equipo investigador tendrá acceso a los datos.  ¿Todas las personas autorizadas a tratar los datos están sujetas a un acuerdo de confidencialidad firmado con el centro?  Sí  No | | |
| **3.7 ¿Se van a ceder datos a terceros?** Sí  No  En caso de cesión se debe especificar:  - los datos cedidos son:  identificados,  seudonimizados,  anonimizados  - a quién se ceden:  - qué datos se ceden:  - con qué finalidad:  - Explique cómo se seudonimizan o se anonimizan los datos:  - Si hay transferencias internacionales: especificar empresa y país (en este caso, se debe solicitar el consentimiento expreso del participante para esta cesión) | | |

| **TRATAMIENTO DE DATOS PERSONALES** | |
| --- | --- |
| **3.8 ¿Se van a realizar grabaciones (audio/vídeo)?**  Recuerde que se necesita consentimiento expreso del interesado para realizar grabaciones, esta información se debe incluir en el documento de consentimiento | Sí  No |
| En caso de grabación, se debe especificar:  En los consentimientos informados se recogerá el consentimiento expreso a la grabación de audio de los grupos focales. Asimismo, al inicio del desarrollo de los grupos focales se recordará que se va a grabar. Los participantes pueden oponerse a dicha grabación en cualquier momento. El contenido de los grupos focales se grabará en una grabadora de audio.  - Dónde se van a conservar, quién tiene acceso y las medidas de seguridad a aplicar:  Los archivos de audio que se extraigan de las grabaciones serán conservados en una carpeta de Sharepoint de Microsoft 365 licencia propiedad de la Universidad San Jorge, al cual solo será posible acceder desde las cuentas corporativas con contraseña. El acceso igualmente solo se realizará desde los ordenadores de los investigadores protegidos con contraseña de acceso. Todos los investigadores del proyecto tendrán acceso a los datos.  - Plazo de conservación de las grabaciones:  Después de la transcripción de las grabaciones, dentro de los 15 días posteriores a cada grabación.  - con qué finalidad:  La finalidad de la obtención y análisis de los datos extraídos en la grabación de audio será únicamente con los fines investigadores indicados en esta memoria. La finalidad de la grabación será la transcripción de esta información posteriormente para ser usada para su posterior análisis.  - si se utilizan aplicaciones informáticas o almacenamiento en la “nube”, se debe indicar quién es el proveedor de servicios y donde está su residencia legal, así como el enlace a su política de privacidad:  Microsoft 365 licencia propiedad de la Universidad San Jorge, Atlas-Ti 24: ATLAS.ti Scientific Software Development GmbH. Bergmannstraße 68. D-10961 Berlin. Germany E-mail: [privacy@atlasti.com](mailto:privacy@atlasti.com) [dataprotection@atlasti.com](mailto:dataprotection@atlasti.com).  <https://atlasti.com/es/legal/politica-de-privacidad> | |
| **3.9 Medidas de seguridad de la información: descripción de los sistemas informáticos que se van a utilizar**  **Se recuerda que los servidores que contengan datos personales deben estar ubicados en el territorio de la UE (RDL 14/2019)** | |
| - Sistema en el que se van a guardar los datos (ordenador personal, servidores corporativos, empresa u organismo externo, proveedor de servicios en la “nube”, etc.)  Los documentos serán guardados a través de uso de un documento Excel ubicado en Sharepoint de Microsoft 365 licencia propiedad de la Universidad San Jorge. El archivo Excel estará protegido por contraseña, se ubicará en una carpeta creada por el investigador principal y compartida únicamente con los miembros del equipo investigador involucrados. Dicha carpeta también estará protegida por contraseña. Y únicamente el investigador principal y los investigadores del proyecto tendrán acceso a esta carpeta.  - Aplicativos que se van a utilizar para el tratamiento de datos (excel, spss, etc.)  Excel en Sharepoint de Microsoft 365 licencia propiedad de la Universidad San Jorge. Los datos se tratarán en el software informático SPPS v.28, Atlas-Ti 24. Todos estarán protegidos con contraseña.  - Si se utilizan aplicaciones informáticas online o almacenamiento en la “nube”, se debe indicar quién es el proveedor de servicios y donde está su residencia legal, así como el enlace a su política de privacidad.  Se utilizará el paquete informático Microsoft 365, siendo el proveedor de éste la Universidad San Jorge, Campus Universitario, Autovía Mudéjar, km. 299, 50830 Villanueva de Gállego, Zaragoza https://www.usj.es/politica-de-privacidad | |
| **3.10 Medidas de seguridad de la información: Dispositivos** | |
| - Indicar si se va a utilizar algún tipo de dispositivo extraíble (USB portátil, disco duro externo, etc.) y si se van a encriptar  No se va a utilizar ningún dispositivo extraíble.  - En el caso de que no se utilicen sistemas informáticos corporativos, indicar si se hacen copias de seguridad.  - Indique las medidas de seguridad para documentos en formato papel (custodia, acceso).  Indicar la persona que custodia los consentimientos informados y encuestas de satisfacción (papel) recogidos de los pacientes y profesionales participantes.  Los documentos en formato papel serán custodiados por el investigador principal, José Lesmes Poveda López, dentro de la taquilla cerrada con llave en la Universidad San Jorge en Villanueva de Gállego (Zaragoza). | |
| **Recomendaciones generales sobre uso de datos**   - No utilizar redes Wifi para transmitir información sensible. - Utilizar contraseñas fuertes y cambiarlas periódicamente. - Encriptar siempre la información sensible que vaya a enviarse por correo electrónico. - Procurar que las versiones de los sistemas operativos y los aplicativos estén siempre actualizados. - En los ordenadores personales, utilizar siempre antivirus y que este actualizado. - No abrir nunca ficheros adjuntos a correos electrónicos en los que no identifiquemos al remitente. - No utilizar redes sociales para comunicar información sensible. - El teléfono móvil es un dispositivo poco seguro para el manejo de información sensible, y los antivirus que se pueden instalar ofrecen poca protección. - Utilizar siempre que se pueda aplicaciones corporativas - El uso de USB u otros dispositivos extraíbles está altamente desaconsejado | |

**4. DESCRIPCIÓN DEL PROYECTO DE INVESTIGACIÓN (completar los campos o adjuntar protocolo completo con la información equivalente)**

| **4.1 Tareas del equipo investigador**  Explicar brevemente quién participa en el estudio, en calidad de qué y qué tareas va a realizar, así como su filiación (puesto de trabajo actual). Se debe presentar el cv y la firma de todos ellos en el [anexo I](#A3)  Si el estudio es multicéntrico debe presentarse un Anexo I por cada centro |
| --- |
| - D. José Lesmes Poveda López. Fisioterapeuta. Docente e investigador de la Universidad San Jorge (Zaragoza). Facultad de Ciencias de la Salud. Grupo de Investigación iPhysio. Investigador Responsable del proyecto. - Dra. Carolina Jiménez Sánchez. Fisioterapeuta. Docente e investigadora de la Universidad San Jorge (Zaragoza). Facultad de Ciencias de la Salud. Grupo de Investigación iPhysio. - Dª. Raquel Lafuente Ureta. Fisioterapeuta. Docente e investigadora de la Universidad San Jorge (Zaragoza). Facultad de Ciencias de la Salud. Grupo de Investigación iPhysio. - Dra. Marta Guarch Rubio. Psicóloga. Docente e investigadora de la Universidad San Jorge (Zaragoza). Facultad de Ciencias de la Salud. Grupo de Investigación IIPOV. - Dr. Juan Francisco Roy Delgado. Psicólogo. Docente e investigador de la Universidad San Jorge (Zaragoza). Facultad de Ciencias de la salud. Grupo de Investigación IIPOV. - Dra. Bárbara Marco-Gómez. Médico psiquiatra. Unidad de corta estancia de Psiquiatría del HURV. Zaragoza. - Dña. Ana Villagrasa Cantín. Supervisora de Enfermería. Unidad de corta estancia de Psiquiatría del HURV. Zaragoza - Dña. Sara Pérez Mansilla. Enfermera especialista en Salud Mental. Unidad de corta estancia de Psiquiatría del HURV. Zaragoza.   A continuación, se detallan las tareas de cada investigador:   \|  \| José Lesmes Poveda López \| Carolina Jiménez Sánchez \| Raquel Lafuente Ureta \| Marta Guarch Rubio \| Juan Francisco Roy Delgado \| Bárbara Marco Gómez \| Ana Villagrasa Cantín \| Sara Pérez Mansilla \| \| --- \| --- \| --- \| --- \| --- \| --- \| --- \| --- \| --- \| \| Diseño del estudio \| **x** \| **x** \| **x** \| **x** \| **x** \| **x** \|  \|  \| \| Tratamiento de los datos \| **x** \|  \| **x** \|  \| **x** \| **x** \|  \|  \| \| Selección de participantes \|  \|  \|  \|  \|  \| **x** \| **x** \| **x** \| \| Acceso a los datos \| **x** \| **x** \| **x** \| **x** \| **x** \| **x** \| **x** \| **x** \| \| Evaluación pre-intervención \| **x** \|  \|  \|  \|  \| **x** \| **x** \| **x** \| \| Evaluación post-intervención \| **x** \|  \|  \|  \|  \| **x** \| **x** \| **x** \| \| Intervención \| **x** \|  \|  \|  \|  \|  \|  \|  \| \| Evaluación cualitativa \|  \| **x** \| **x** \|  \|  \| **x** \| **x** \| **x** \| \| Análisis de resultados \|  \| **x** \| **x** \| **x** \| **x** \|  \|  \|  \| \| Interpretación de los resultados \|  \| **x** \| **x** \| **x** \| **x** \|  \|  \|  \| \| Elaboración y difusión de los resultados de la investigación \| **x** \| **x** \| **x** \| **x** \| **x** \| **x** \| **x** \| **x** \| |
| **4.2 Justificación del estudio: Antecedentes, estado actual del tema, relevancia** (Citar las referencias bibliográficas en el apartado siguiente) |
| La Organización Mundial de la Salud(1) indica que la salud mental es el estado de salud que permite afrontar a las personas los momentos de estrés con capacidades y habilidades, que es un derecho humano fundamental y elemento necesario para el desarrollo comunitario. Su merma conlleva la enfermedad mental, caracterizada por situaciones de angustia, discapacidad funcional, mayor dificultad para las actividades de la vida diaria básicas e instrumentales, sedentarismo y atrofia muscular, mala calidad de vida, baja fuerza muscular y fatiga, dolor, cambios en el tono muscular, deterioro cognitivo y afectivo, aislamiento social, estigmatización, baja laboral y riesgo de autolesión y muerte prematura, además de generar sobrecarga asistencial en los servicios públicos de salud y aumento de costos(2).  La prevalencia de problemas de salud mental en España alcanza el 27,4 % en la población global, siendo el trastorno depresivo mayor la enfermedad mental con mayor tasa de incidencia en la población adulta (4,1% de la población adulta española y el 5% en la población adulta a nivel mundial), seguidos de los trastornos de ansiedad, trastornos de personalidad, trastornos psicóticos, alteraciones cognitivas, anorexia nerviosa, bulimia y los trastornos obsesivos compulsivos(3). Tras el primer año después de la pandemia del Covid-19 las enfermedades mentales aumentaron su prevalencia un 25% a nivel mundial, principalmente los trastornos depresivos y de ansiedad(4).  El trastorno depresivo mayor, así clasificado según la clasificación internacional de enfermedades en su décima versión (CIE-10)(5) y por el manual de trastornos mentales DSM-5 de la Asociación de Psiquiatría de EE.UU. (6), es un trastorno del estado de ánimo, caracterizado por una tristeza profunda y pérdida de interés por cualquier actividad de forma mantenida, como mínimo durante dos semanas, suponiendo un cambio en el estado basal de la persona, no explicable por otra enfermedad, por el consumo de tóxicos o farmacológico. Es una enfermedad que se asocia con una pérdida de calidad de vida muy importante, acompañada de síntomas tanto psicológicos (anhedonia, anergia, alteración del sueño y del apetito) como físicos por el deterioro funcional, siendo así la principal causa mundial de discapacidad(7), afectando más a las mujeres(8) y a la población con menores ingresos económicos(9,10), causando un grave sufrimiento y alteración sobre las actividades laborales, familiares y sociales, y que como consecuencia mayor sería el suicidio(11,12). Es una enfermedad que puede ser de episodio único o recurrente durante más de dos años, con una severidad de leve, moderada o grave(13). Todos estos problemas afectan de forma significativa a la población, lo que requiere de un enfoque integral para su manejo.  También debemos tener en cuenta el proceso de envejecimiento de la población, actualmente un 19% de población mayor de 65 años en España, y con una previsión a más del 25% en 10 años(14). En esta población, el estado de mala salud mental provoca un mayor riesgo de comorbilidad y tasa de discapacidad(15), soledad, estigmatización e institucionalización(16,17), lo que incide en el mayor riesgo de mortalidad(18,19). Los condicionantes del envejecimiento hacen que esta población requiera de un seguimiento más exhaustivo por la gravedad de sus repercusiones y el aumento de gasto y uso de recursos sanitarios(20).  Así pues, debe existir un programa de promoción, intervención y educación para la salud en este grupo de personas con trastorno depresivo mayor para favorecer su recuperación, bajo una coordinación interdisciplinar, siendo necesaria la formación y promoción de profesionales sanitarios especializados que puedan mejorar esta situación(8,21,22). La OMS considera la promoción de la salud mental un eje de gran interés e insta a las organizaciones y estados a que apliquen planes de acción basados en tres vías de transformación: dar más valor a la salud mental, actuar sobre la condición física, social y económica, y fortalecer finalmente una red comunitaria de servicios de apoyo(1).  El trastorno depresivo mayor es una enfermedad multifactorial, donde influyen factores genéticos y ambientales(23). La presencia de estos factores conlleva una disminución de los neurotransmisores: serotonina, noradrenalina y dopamina. Esta disfunción está mediada por una hiperactividad del eje hipotálamo-hipofisario-adrenal (HHA) encargada del control del estrés y de la presencia de cortisol, y una disminución del volumen morfológico del hipocampo por disminución de dendritas y redes neuronales(24,25). El trastorno depresivo mayor se considera una enfermedad inflamatoria debido al aumento de citoquinas proinflamatorias en sangre en estos pacientes, causada por esa activación del eje HHA(7).  Los tratamientos habituales del trastorno depresivo mayor son de enfoque interdisciplinar, incluyendo principalmente el pilar médico(26–28) (atención primaria y especializada), el farmacológico(29–31), el psicoterápico(32,33), y en menor medida, la fisioterapia(34,35). La puerta de entrada al sistema de asistencia se encuentra en la atención primaria, desde donde el profesional médico realiza el primer diagnóstico clínico y toma de decisiones sobre el tratamiento farmacológico necesario, valora el nivel de severidad de sus síntomas a través de las herramientas de cribaje y escalas de evaluación, para además tener la información para el seguimiento y poder evaluar la necesidad de pasar a otro nivel asistencial. El apoyo del médico psiquiatra será necesario en el seguimiento a largo plazo de los pacientes cuando la primera línea de acción no sea efectiva, y determinar la derivación a profesionales de la psicología, la terapia ocupacional o la fisioterapia. Importante es el papel de la enfermería de salud mental, que además de ser una especialidad reconocida en los cuidados de estos pacientes, sirve de seguimiento y análisis del proceso de enfermedad en apoyo de la medicina. En general la farmacología puede tener un aceptable buen resultado, pero la falta de adherencia y control sobre su uso no siempre es la adecuada, y en algunos casos los efectos adversos que provocan llevan al paciente a un estado de somnolencia que dificultad su independencia(36). En cuanto la psicoterapia, las técnicas de terapia cognitivo-conductual, de resolución de problemas o revisión de vida, entre otras, son comunes para tratar principalmente los síntomas afectivos y emocionales más afectados en el trastorno depresivo mayor(32).  La Confederación Mundial de Fisioterapia (WCPT) define la fisioterapia como un servicio que presta a la población para desarrollar, mantener y restablecer el máximo movimiento y capacidad funcional a lo largo de la vida(37). Esta atención se presta en circunstancias en que el movimiento y la función se ven afectados por el envejecimiento, las lesiones, el dolor o la enfermedad, entendiendo que el movimiento es fundamental para la salud. Los fisioterapeutas se ocupan de identificar el nivel de calidad de vida de las personas y plantear un tratamiento a través del movimiento que promocione, prevenga, intervenga y rehabilite sus capacidades mermadas por la enfermedad, la cual no solo abarca la esfera física, si no también, la psicológica, emocional y social, haciendo hincapié en la necesaria adición de la educación para la salud(38).Es competencia de la fisioterapia mantenerse alineada con las necesidades de la población, e investigar y atender aquellas pautas sanitarias que sean claves para el desarrollo de la salud de su entorno, siempre bajo el estricto cumplimiento de su código de principios éticos. La fisioterapia es una profesión sanitaria independiente, pero que necesita integrarse en un equipo interdisciplinar para poder detectar, planificar y asegurar todos los factores que predisponen a la mejoría de la salud mental de los pacientes. Dentro de sus funciones resaltarían(39):   - Realizar entrevistas con profesionales y pacientes en salud mental para conocer las necesidades, objetivos y expectativas del tratamiento. - Realizar valoraciones generales del estado de salud, y específicas en fisioterapia de las capacidades musculoesqueléticas, respiratorias y circulatorias. - Diseñar un plan estratégico de tratamiento acorde a las necesidades del servicio y tipología de estancia en planta de psiquiatría, fomentando el trabajo grupal entre varios pacientes, y en su caso, con intervenciones individuales que sean precisas. - Detectar los factores de riesgo que pueden alterar el estado de salud del paciente en servicio de psiquiatría, que disminuyen la calidad y esperanza de vida. - Realizar un plan estratégico de educación para la salud, buscando fomentar hábitos saludables que favorezcan el estado de ánimo y las fortalezas de los pacientes. - Realizar revisiones periódicas del estado de salud de los pacientes. - Realizar reuniones de equipo interdisciplinar.   Evaluar la efectividad del tratamiento en todas sus dimensiones, tanto sobre la patología física como mental a través de la investigación de técnicas y procedimientos basados en la ciencia, con la finalidad de poder asentar un razonamiento clínico con evidencia que permita defender la necesidad del tratamiento fisioterapéutico.  La fisioterapia en salud mental, y específicamente para el paciente con trastorno depresivo mayor, permite trabajar de forma integral sobre el paciente, mejorando la conexión cuerpo y mente a través del movimiento, haciendo a la persona más consciente de sus capacidades y necesidades, dando herramientas de fortalecimiento y confrontación con las dificultades que pueda encontrar en la realización de tareas en su día a día(40). Muchos síntomas físicos que pueden presentarse derivados del trastorno depresivo mayor se podrán tratar para empoderar al paciente y permitirle ser más activo en la superación de los eventos estresantes, además de que el movimiento podrá mejorar el ánimo a través de la liberación de endorfinas(41). El tratamiento del dolor crónico asociado a estados de depresión ha tenido efectos clínicamente significativos(42–44), que a su vez han incidido en la mejora funcional de las personas afectadas, así pues, la realización de ejercicio terapéutico a través de la fisioterapia asegura un trato profesional y alineado con el resto de los sanitarios que mejorará el estado emocional y físico(35).  ***Justificación del proyecto***  Debido a la alta prevalencia del trastorno depresivo mayor, su grave repercusión sobre la salud del paciente adulto, cómo afecta a la comunidad y al sistema sanitario, sería pertinente explorar los tratamientos de fisioterapia de ejercicio físico terapéutico y educación sanitaria y evaluar su efectividad sobre su calidad de vida y estado de salud, como un complemento a los tratamientos psiquiátricos y/o psicológicos ya presentes, en el ámbito de una unidad de psiquiatría de hospitalización de corta estancia. |
| **4.3 Bibliografía** (debe estar referenciada en el texto anterior) |
| 1. Informe Mundial Sobre Salud Mental Transformar la Salud Mental para Todos. Panorama General. Geneva: World Health Organization; 2022.  2. Modelo de atención a las personas con enfermedad mental grave. 1a. ed. Madrid: Instituto de Mayores y Servicios Sociales; 2007.  3. Informe Salud mental en datos: prevalencia de los problemas de salud y consumo de psicofármacos y fármacos relacionados a partir de los registros clínicos de atención primaria. Ministerio de Sanidad de España; 2020.  4. Santomauro DF, Mantilla Herrera AM, Shadid J, Zheng P, Ashbaugh C, Pigott DM, et al. Global prevalence and burden of depressive and anxiety disorders in 204 countries and territories in 2020 due to the COVID-19 pandemic. The Lancet. noviembre de 2021;398(10312):1700-12.  5. CIE-10-ES: Clasificación Internacional de Enfermedades - 10.Œ revisión : modificación clínica. 2a ed., enero 2018. Madrid: Ministerio de Sanidad, Servicios Sociales e Igualdad; 2018.  6. Manual diagnóstico y estadístico de los trastornos mentales: DSM-5. 5.^a^ ed., 2.^a^ reimp. Buenos Aires [etc.]: Editorial Médica Panamericana; 2016.  7. Beurel E, Toups M, Nemeroff CB. The Bidirectional Relationship of Depression and Inflammation: Double Trouble. Neuron. julio de 2020;107(2):234-56.  8. Dotson VM, Hsu FC, Langaee TY, McDonough CW, King AC, Cohen RA, et al. Genetic Moderators of the Impact of Physical Activity on Depressive Symptoms. J Frailty Aging. 2016;5(1):6-14.  9. Maier A, Riedel-Heller SG, Pabst A, Luppa M. Risk factors and protective factors of depression in older people 65+. A systematic review. Bayer A, editor. PLoS ONE. 13 de mayo de 2021;16(5):e0251326.  10. Cleary JL, Fang Y, Zahodne LB, Bohnert ASB, Burmeister M, Sen S. Polygenic Risk and Social Support in Predicting Depression Under Stress. AJP. 1 de febrero de 2023;180(2):139-45.  11. Ribeiro JD, Huang X, Fox KR, Franklin JC. Depression and hopelessness as risk factors for suicide ideation, attempts and death: meta-analysis of longitudinal studies. Br J Psychiatry. mayo de 2018;212(5):279-86.  12. O’Connor SJ, Hewitt N, Kuc J, Orsini LS. Predictors and Risk Factors of Treatment-Resistant Depression: A Systematic Review. J Clin Psychiatry [Internet]. 13 de noviembre de 2023 [citado 28 de agosto de 2024];85(1). Disponible en: https://www.psychiatrist.com/jcp/predictors-risk-factors-treatment-resistant-depression-systematic-review/  13. Dávila Hernández A, González González R, Liangxiao M, Xin N. Estudio sinomédico de la fisiopatología de la depresión. Revista Internacional de Acupuntura. enero de 2016;10(1):9-15.  14. Mayores a un clic - Instituto de Mayores y Servicios Sociales [Internet]. [citado 28 de agosto de 2024]. Disponible en: https://imserso.es/espacio-mayores/estadisticas/mayores-un-clic  15. Boehlen FH, Herzog W, Maatouk I, Saum KU, Brenner H, Wild B. Treatment preferences of elderly patients with mental disorders. Z Gerontol Geriatr. febrero de 2016;49(2):120-5.  16. Boström G, Conradsson M, Hörnsten C, Rosendahl E, Lindelöf N, Holmberg H, et al. Effects of a high-intensity functional exercise program on depressive symptoms among people with dementia in residential care: a randomized controlled trial. Int J Geriatr Psychiatry. agosto de 2016;31(8):868-78.  17. Conradsson M, Littbrand H, Lindelof N, Gustafson Y, Rosendahl E. Effects of a high-intensity functional exercise programme on depressive symptoms and psychological well-being among older people living in residential care facilities: A cluster-randomized controlled trial. Aging Ment Health. julio de 2010;14(5):565-76.  18. Zhang Z, Jackson SL, Gillespie C, Merritt R, Yang Q. Depressive Symptoms and Mortality Among US Adults. JAMA Netw Open. 9 de octubre de 2023;6(10):e2337011.  19. Von Below A, Hällström T, Sundh V, Björkelund C, Hange D. Association between anxiety and depression and all-cause mortality: a 50-year follow-up of the Population Study of Women in Gothenburg, Sweden. BMJ Open. noviembre de 2023;13(11):e075471.  20. Underwood M, Lamb S, Eldridge S, Sheehan B, Slowther A, Spencer A, et al. Exercise for depression in care home residents: a randomised controlled trial with cost-effectiveness analysis (OPERA). Health Technol Assess [Internet]. mayo de 2013 [citado 21 de abril de 2024];17(18). Disponible en: https://www.journalslibrary.nihr.ac.uk/hta/hta17180/  21. Price RB, Duman R. Neuroplasticity in cognitive and psychological mechanisms of depression: an integrative model. Mol Psychiatry. marzo de 2020;25(3):530-43.  22. Fox ME, Lobo MK. The molecular and cellular mechanisms of depression: a focus on reward circuitry. Mol Psychiatry. diciembre de 2019;24(12):1798-815.  23. Pitsillou E, Bresnehan SM, Kagarakis EA, Wijoyo SJ, Liang J, Hung A, et al. The cellular and molecular basis of major depressive disorder: towards a unified model for understanding clinical depression. Mol Biol Rep. enero de 2020;47(1):753-70.  24. Hussenoeder FS, Jentzsch D, Matschinger H, Hinz A, Kilian R, Riedel-Heller SG, et al. Depression and quality of life in old age: a closer look. Eur J Ageing. marzo de 2021;18(1):75-83.  25. Jung ES, Choi YY, Lee KH. Effects of Integrative Cognitive Function Improvement Program on Cognitive Function, Oral Health, and Mental Health in Older People: A Randomized Clinical Trial. International Journal of Environmental Research and Public Health [Internet]. 2022;19(21). Disponible en: https://www.scopus.com/inward/record.uri?eid=2-s2.0-85141552646&doi=10.3390%2fijerph192114339&partnerID=40&md5=ffbce39036c5a7ce3a8bbb858568c5ed  26. Sukhato K, Lotrakul M, Dellow A, Ittasakul P, Thakkinstian A, Anothaisintawee T. Efficacy of home-based non-pharmacological interventions for treating depression: a systematic review and network meta-analysis of randomised controlled trials. BMJ Open. julio de 2017;7(7):e014499.  27. Goodwin GM, Stein DJ. Generalised Anxiety Disorder and Depression: Contemporary Treatment Approaches. Adv Ther. septiembre de 2021;38(S2):45-51.  28. Baba H. Treatment strategy for late‐life depression. PCN Reports. junio de 2023;2(2):e91.  29. Höppner J, Schulz M, Irmisch G, Mau R, Schläfke D, Richter J. Antidepressant efficacy of two different rTMS procedures. High frequency over left versus low frequency over right prefrontal cortex compared with sham stimulation. Eur Arch Psychiatry Clin Neurosci. abril de 2003;253(2):103-9.  30. Kishi T, Ikuta T, Sakuma K, Okuya M, Hatano M, Matsuda Y, et al. Antidepressants for the treatment of adults with major depressive disorder in the maintenance phase: a systematic review and network meta-analysis. Mol Psychiatry. enero de 2023;28(1):402-9.  31. Nuñez NA, Joseph B, Pahwa M, Kumar R, Resendez MG, Prokop LJ, et al. Augmentation strategies for treatment resistant major depression: A systematic review and network meta-analysis. Journal of Affective Disorders. abril de 2022;302:385-400.  32. Cuijpers P, Karyotaki E, Eckshtain D, Ng MY, Corteselli KA, Noma H, et al. Psychotherapy for Depression Across Different Age Groups: A Systematic Review and Meta-analysis. JAMA Psychiatry. 1 de julio de 2020;77(7):694.  33. Bhattacharya S, Kennedy M, Miguel C, Tröger A, Hofmann SG, Cuijpers P. Effect of psychotherapy for adult depression on self-esteem: A systematic review and meta-analysis. Journal of Affective Disorders. marzo de 2023;325:572-81.  34. Lialy HE, Mohamed MA, AbdAllatif LA, Khalid M, Elhelbawy A. Effects of different physiotherapy modalities on insomnia and depression in perimenopausal, menopausal, and post-menopausal women: a systematic review. BMC Women’s Health. 8 de julio de 2023;23(1):363.  35. Noetel M, Sanders T, Gallardo-Gómez D, Taylor P, Del Pozo Cruz B, Van Den Hoek D, et al. Effect of exercise for depression: systematic review and network meta-analysis of randomised controlled trials. BMJ. 14 de febrero de 2024;e075847.  36. Hennessy S, Leonard C, Gagne J, Flory J, Han X, Brensinger C, et al. Pharmacoepidemiologic Methods for Studying the Health Effects of Drug–Drug Interactions. Clin Pharma and Therapeutics. enero de 2016;99(1):92-100.  37. Organización Internacional de Fisioterapia en Salud Mental (IOPTMH) \| Fisioterapia mundial [Internet]. [citado 28 de agosto de 2024]. Disponible en: https://world.physio/es/subgroups/mental-health  38. Breitve MH, Hynninen MJ, Kvåle A. The effect of psychomotor physical therapy on subjective health complaints and psychological symptoms. Physiother Res Int. diciembre de 2010;15(4):212-21.  39. International Organization of Physical Therapy in Mental Health [Internet]. [citado 28 de agosto de 2024]. Disponible en: https://www.ioptmh.org/  40. Ayaz EY, Dincer B, Mete E, Benli RK, Cinbaz G, Karacan E, et al. Evaluating the impact of aerobic and resistance green exercises on the fitness, aerobic and intrinsic capacity of older individuals. Arch Gerontol Geriatr. marzo de 2024;118:105281.  41. Schuch FB, Vancampfort D. Physical activity, exercise, and mental disorders: it is time to move on. Trends Psychiatry Psychother [Internet]. 2021 [citado 25 de agosto de 2023]; Disponible en: https://www.scielo.br/j/trends/a/jCRvs9LQq8ycmLwLysGBRFM/?lang=en  42. Fernández-Pérez P, Leirós-Rodríguez R, Marqués-Sánchez MP, Martínez-Fernández MC, De Carvalho FO, Maciel LYS. Effectiveness of physical therapy interventions in women with dyspareunia: a systematic review and meta-analysis. BMC Women’s Health. 24 de julio de 2023;23(1):387.  43. Kannan P, Lam HY, Ma TK, Lo CN, Mui TY, Tang WY. Efficacy of physical therapy interventions on quality of life and upper quadrant pain severity in women with post-mastectomy pain syndrome: a systematic review and meta-analysis. Qual Life Res. abril de 2022;31(4):951-73.  44. Cohen-Biton L, Buskila D, Nissanholtz-Gannot R. Review of Fibromyalgia (FM) Syndrome Treatments. IJERPH. 24 de septiembre de 2022;19(19):12106.  45. Hernandez G, Garin O, Pardo Y, Vilagut G, Pont À, Suárez M, et al. Validity of the EQ–5D–5L and reference norms for the Spanish population. Qual Life Res. septiembre de 2018;27(9):2337-48.  46. Lobo A, Chamorro L, Luque A, Dal-Ré R, Badia X, Baró E. Validación de las versiones en español de la Montgomery-Asberg Depression Rating Scale y la Hamilton Anxiety Rating Scale para la evaluación de la depresión y de la ansiedad. Medicina Clínica. enero de 2002;118(13):493-9.  47. Hjermstad MJ, Fayers PM, Haugen DF, Caraceni A, Hanks GW, Loge JH, et al. Studies Comparing Numerical Rating Scales, Verbal Rating Scales, and Visual Analogue Scales for Assessment of Pain Intensity in Adults: A Systematic Literature Review. Journal of Pain and Symptom Management. junio de 2011;41(6):1073-93.  48. Scholz U, Gutiérrez Doña B, Sud S, Schwarzer R. Is General Self-Efficacy a Universal Construct?1. European Journal of Psychological Assessment. septiembre de 2002;18(3):242-51.  49. Gómez Ramírez OJ, Carrillo González GM, Cárdenas DC. Encuesta de satisfacción con el cuidado de la salud en las personas con enfermedad crónica. eglobal. 27 de septiembre de 2016;15(4):321.  50. Gobierno de Aragón. Informe de prevalencia de depresión en Aragón 2022. Zaragoza: Gobierno de Aragón; 2022..  51. Salk RH, Hyde JS, Abramson LY. Gender differences in depression in representative national samples: Meta-analyses of diagnoses and symptoms. Psychological Bulletin. agosto de 2017;143(8):783-822. |
| **4.4 Hipótesis** (afirmación que se pretende demostrar) |
| La intervención con un programa de fisioterapia basado en ejercicio físico terapéutico y educación sanitaria, junto a los tratamientos habituales en pacientes adultos con trastorno depresivo mayor, en una unidad psiquiátrica de corta estancia, muestra efectividad comparando valores pre y post intervención sobre la calidad de vida y estado de salud, medida a través de escalas validadas. |
| **4.5 Objetivos** |
| ***Objetivo principal***  Analizar la efectividad de un programa de intervención en fisioterapia basado en ejercicio físico terapéutico y educación sanitaria, sobre su estado de salud general, mental y físico, y calidad de vida de las personas adultas con trastorno depresivo mayor durante su ingreso en una unidad de corta estancia de Psiquiatría del Hospital Royo Villanova de Zaragoza, medido a través de la escala de calidad de vida EQ-5D-3L(45).  ***Objetivos secundarios***  Analizar la efectividad sobre los síntomas de la depresión clínica, medido a través de la escala Montgomery-Asberg Depression Rating Scale en su versión validada al español(46).  Analizar la efectividad el del síntoma de dolor, medido a través de la escala NRS(47).  Analizar la efectividad sobre la autoeficacia, medido a través de la Escala de Autoeficacia General(48).  Analizar el nivel de satisfacción con el programa de intervención de fisioterapia, medido a través de la Encuesta de satisfacción con el cuidado de la salud para personas con enfermedad crónica (GCPC-UN-ESU)(49).  Describir a través de grupos focales cualitativos las experiencias y percepciones de los participantes con la enfermedad y en el programa de intervención de fisioterapia, así como las percepciones sobre el manejo de su proceso, y las barreras y facilitadores que los tratamientos, incluido el propuesto en este proyecto, suponen para ellos.  Describir a través de grupos focales cualitativos las percepciones de los profesionales que integran el equipo profesional (sanitario y no sanitario) de Psiquiatría del Hospital Royo Villanova de Zaragoza en relación al programa de intervención de fisioterapia, así como las percepciones que han tenido sobre el manejo de la enfermedad, las barreras y facilitadores, incluido el propuesto en este proyecto, en relación a los participantes ingresados en su unidad. |

| **4.6 Metodología** (se deben detallar **todos** los campos siguientes**):**  Diseño del estudio  Participantes: criterios de inclusión/exclusión; modo de reclutamiento (quién y cómo realiza el contacto inicial con los participantes, presentar material de difusión del estudio, si lo hay), tamaño muestral (y su justificación), aleatorización (si procede)  Fuentes de información: variables detalladas (datos a recoger), origen de los datos, cuándo y cómo se recogen, a qué periodo de tiempo se refieren.  Procedimientos: detallar de forma diferenciada los procedimientos puramente asistenciales de los propios de la investigación, presentar encuestas o formularios que se vayan a utilizar (link en caso de ser encuestas online), valoración del riesgo de los procedimientos experimentales y medidas para minimizarlo.  Análisis estadístico  Consideración de la perspectiva de género: detallar las medidas adoptadas para que los resultados del estudio puedan reflejar posibles diferencias por sexo/género.  Limitaciones del estudio  En caso de muestras biológicas: detallar tipo y número de muestras, cómo se recogen, dónde y quién las analiza, cuándo se destruyen (o destino final) |
| --- |
| ***Diseño del estudio***  Se va a llevar a cabo un estudio de diseño mixto concurrente anidado de dominancia cuantitativa, siguiendo los criterios descritos en la guía Métodos Mixtos de Investigación (MMARS). El estudio cuantitativo será un estudio piloto cuasiexperimental de diseño pre-post. El presente estudio cuenta además con un diseño adicional cualitativo narrativo, en el que se llevarán a cabo grupos focales, para el análisis de las experiencias con la intervención, así como las percepciones sobre el manejo de su proceso, y las barreras y facilitadores que los participantes han tenido durante la realización del programa de intervención, y por otro lado en relación a las percepciones de los profesionales que integran el equipo profesional (sanitario y no sanitario) de Psiquiatría del Hospital Royo Villanova de Zaragoza en relación al programa de intervención propuesto y como perciben que ha influido sobre el manejo de la enfermedad, las barreras y facilitadores, sobre los pacientes ingresados en su unidad.  El estudio cuenta con la autorización del jefe del servicio de psiquiatría del Sector I del Servicio Aragonés de Salud y de la Gerencia-Dirección Médica del Hospital Universitario Royo Villanova de Zaragoza (ANEXOS I y II). Asimismo, se ha establecido un convenio de colaboración entre la USJ y Gerencia-Dirección Médica del Hospital Royo Villanova. Se ha obtenido informe favorable del comité de ética de la Universidad San Jorge  Nº 38/3/24-25.  ***Población***  La muestra estará compuesta por pacientes voluntarios consecutivos ingresados en la Unidad de corta estancia de Psiquiatría del Hospital Royo Villanova (Zaragoza) que cumplan los siguientes criterios de selección:  - Criterios de inclusión:   - Mayores de 18 años. - Ingresados en la planta de psiquiatría del Hospital Royo Villanova. - Diagnóstico de trastorno depresivo mayor como enfermedad mental por parte de profesional médico. - En tratamiento habitual médico, psicológico y farmacológico de su enfermedad. - No necesidad de supervisión y control por personal profesional de la unidad de psiquiatría durante la toma de datos e intervención.   - Criterios de exclusión:   - Presencia comórbida de enfermedad física o mental cuyas características clínicas y/o severidad impida la comprensión y/o el seguimiento de las intervenciones de fisioterapia. - Presencia de disfuncionalidad o discapacidad física o mental que impidan o sean contraindicaciones totales o parciales de las técnicas de fisioterapia. - Incapacidad legal. - Embarazo.   - Criterios de abandono:   - Deseo expreso del sujeto de abandonar el estudio. - Asistencia menor al 80% de las sesiones de intervención de fisioterapia. - Lesión que impida continuar con las sesiones. - Nueva enfermedad cuyo diagnóstico y/o severidad impida continuar con el estudio o *exitus*.   Para un diseño de un solo grupo se ha realizado el cálculo del tamaño muestral en función de un tamaño del efecto moderado por ser un estudio piloto (d = 0.5), con un nivel de significancia de 0.05 y una potencia de 0.80. También se ha tenido en cuenta una tasa de abandonos del 25%. De esta forma, se necesitaría alrededor de 40 participantes para detectar un cambio significativo teniendo en cuenta que cumplan los criterios de selección donde la estancia media de ingreso en la unidad de corta estancia de Psiquiatría del Hospital Royo Villanova es de 3 semanas y tiene una capacidad máxima de 25 pacientes.  La muestra (8 personas) que formará parte del grupo focal en la evaluación cualitativa de profesionales que integran el equipo profesional (sanitario y no sanitario) de Psiquiatría del Hospital Royo Villanova de Zaragoza constará de participantes voluntarios y deberá cumplir los siguientes criterios de selección:  - Criterios de inclusión:   - Profesionales sanitarios y no sanitarios de la unidad de corta estancia de Psiquiatría del Hospital Royo Villanova de Zaragoza.   - Criterios de exclusión:   - Profesionales de la unidad que no hayan estado activos (contrato laboral único, fijo o temporal) laboralmente durante toda la intervención en fisioterapia.   - Criterios de abandono:   - Deseo expreso del sujeto de abandonar el estudio. - Baja laboral o *exitus*. - Cambio de puesto a otra unidad o centro sanitario.   ***Evaluación***  *Evaluación cuantitativa*  Se realizará una evaluación inicial antes del comienzo de la intervención y otra al finalizar dicha intervención, siempre antes del alta hospitalaria. Los datos de la evaluación pre-intervención se recogerán los primeros días de cada ingreso, para cada paciente que quiera participar voluntariamente, y los datos de la evaluación post-intervención se tomarán los días previos a su alta hospitalaria. Esta recogida de datos se realizará a lo largo de las semanas que sean precisas hasta alcanzar el tamaño de la muestra. Como se ha indicado anteriormente, la media de estancia hospitalaria puede ser de 3 semanas, dentro de las cuales se hará la pre-post evaluación y la intervención del programa.   - Mediciones iniciales-variables de control   Datos sociodemográficos: edad, género, profesión.  Datos clínicos: talla, peso, índice de masa corporal, diagnóstico médico, tratamiento farmacológico, otros tratamientos no farmacológicos.   - Variable principal   CALIDAD DE VIDA: **EuroQuol-5D-3L versión en español (EQ5D-3L Spanish version)**  El cuestionario de salud EQ-5D-3L es un cuestionario que se puede autoadministrar o realizar a través de entrevista, que permite conocer el estado de salud en relación a varias dimensiones del paciente (movilidad, dolor, salud mental…). Consta de 5 preguntas con 3 opciones de respuesta (buena salud=1, algún problema=2 o problema de salud=3) donde se codifican las respuestas y se aplican coeficientes según respuestas para alcanzar un valor de referencia; y por otro lado consta de una escala visual analógica vertical de 20 centímetros, con valores de 0=peor estado de salud a 100=mejor estado de salud, donde el paciente indica el valor actual de salud percibida. Es una herramienta validada y muy usado en salud e investigación.   - Variables específicas   DEPRESIÓN: **Montgomery-Asberg Depression Rating Scale (MADRS) versión validada al español**  Esta escala es de uso clínico para la detección y severidad del trastorno depresivo mayor. Consta de 10 preguntas que se realizan al entrevistado sobre síntomas cognitivos y afectivo/emocionales con valores de respuesta entre el 0 y el 6 (0=menor nivel de sintomatología y 6=máximo nivel de sintomatología). Su interpretación será 0-6 puntos no depresión, 7-19 puntos depresión leve, 20-34 puntos depresión moderada y 35-60 puntos depresión grave.  DOLOR: **Escala NRS**  La escala NRS para la valoración del dolor es una escala que mide la intensidad del dolor que presenta el paciente. Es una escala numérica donde se le pide al paciente valorar su dolor entre 0 y 10. Los valores reportados se clasifican: no dolor=0; dolor leve=1,2; dolor moderado=3-5; dolor intenso=6-8; dolor insoportable=9-10.  AUTOEFICACIA: **Escala de autoeficacia general (General Self-efficacy scale)**  La versión española de la Escala de Autoeficacia general (General Self-efficacy Scale) consiste en un cuestionario de 10 preguntas que permiten medir la percepción de la persona sobre sus propias capacidades para manejar su vida ante situaciones estresantes. Ante cada pregunta el entrevistado debe contestar siguiendo una escala Likert donde se puntúa la respuesta como 1 totalmente en desacuerdo y 5 totalmente de acuerdo. La puntuación entre 27 y 38 puntos se considera una media de autoeficacia. A mayor puntuación mayor percepción de autoeficacia.  SATISFACCIÓN: **Encuesta de satisfacción con el cuidado de la salud para personas con enfermedad crónica (GCPC-UN-ESU)**  La encuesta de satisfacción con el cuidado de la salud para personas con enfermedad crónica (GCPC-UN-ESU) es una herramienta validad para medir el nivel de satisfacción. Contiene 19 ítems con 4 dimensiones: los cuidados, la educación en salud, la calidad del servicio prestado y el nivel de fidelización con el servicio. Cada ítem tiene una puntuación entre 1 (nada satisfecho) y 5 (enormemente satisfecho). A mayor puntuación, mayor satisfacción. Se medirá solamente en la valoración post intervención y de forma anónima.  *Evaluación cualitativa*  Se llevarán a cabo grupos focales diferentes con dos grupos de población diferentes:   - Grupos focales solo con pacientes participantes con trastorno depresivo mayor que hayan realizado la intervención en la unidad de corta estancia del Hospital Universitario Royo Villanova de Zaragoza. - Grupos focales solo con profesionales participantes de la unidad de corta estancia de Psiquiatría del Hospital Universitario Royo Villanova de Zaragoza.   Se le pedirá a cada grupo de participantes que colaboren en la realización de un grupo focal. Se realizarán varios grupos focales con estos perfiles, cada uno de ellos con 7-8 pacientes participantes (dependiendo de la muestra final reclutada) y 8 profesionales participantes tratando de buscar la heterogeneidad dentro de cada grupo y la homogeneidad entre grupos focales. Los grupos focales serán solo de pacientes o solo de profesionales.  Los grupos focales con pacientes se llevarán a cabo de forma secuencial tras el alta hospitalaria que hayan realizado la intervención y la evaluación pre-post cuantitativa.  Los grupos focales con profesionales se llevarán a cabo dentro de las dos semanas posteriores a la finalización de la fase cuantitativa, la cual finalizará una vez alcanzado el tamaño de la muestra necesario. La guía de preguntas para los grupos focales de pacientes versará sobre la vivencia con la enfermedad, las percepciones en el manejo de su proceso, y las barreras y facilitadores que han tenido durante la realización del programa de intervención (ANEXO III). La guía de preguntas para los grupos focales de profesionales versará sobre las percepciones sobre el manejo de la enfermedad de los pacientes, y las barreras y facilitadores que han podido observar durante la realización del programa de intervención.  Cada grupo focal será dirigido por un investigador, mientras que otro investigador será el encargado de tomar las notas de campo e impresiones que vaya captando de los profesionales participantes durante el desarrollo del grupo focal, los dos estarán capacitados en investigación cualitativa y no habrán tenido relación previa con los participantes. Ambos investigadores realizarán un proceso previo de posicionamiento del investigador o "bracketing", con el objetivo de asegurar que sus conocimientos y experiencias previas, creencias y motivación para la investigación no influyan en la recopilación y análisis de datos.  El grupo focal tendrá una duración de 1h aproximadamente basado en una guía de preguntas (que se adjunta en el ANEXO III) y será grabado mediante grabadora, tras el consentimiento firmado de cada uno de los profesionales participantes.  ***Intervención***  Todos los participantes recibirán la misma intervención basada en la evidencia científica en la unidad de corta estancia de Psiquiatría del Hospital Royo Villanova de Zaragoza. La intervención será grupal, con un número máximo de 8 pacientes por fisioterapeuta, y se llevará a cabo en un tiempo de 3 semanas, realizándose 2 sesiones semanales de aproximadamente 45 minutos. Se diferenciarán dos partes en cada sesión:   - Parte de ejercicio terapéutico basado en movilidad articular activa, ejercicios de fuerza con propio peso y/o bandas elásticas, ejercicios de equilibrio y ejercicios de relajación muscular progresiva. Será necesario el uso de esterillas, sillas y bandas elásticas. - Parte de educación sanitaria dirigidos a mejorar el conocimiento de su estado físico derivado de la enfermedad mental y mejorar la autogestión de sus capacidades físicas para mejor autoeficacia y destrezas motrices.   ***Análisis de los datos***  El análisis estadístico de los datos cuantitativos se realizará con el software IBM-SPSS Statistics versión 29. Los datos se expresarán como media y desviación estándar o mediana y rango intercuartílico. Se utilizará la prueba de normalidad de Shapiro-Wilk. Se llevarán a cabo ANOVA simples y/o de medidas repetidas para contrastar las diferencias entre puntuaciones pre- y post-test, en caso de que no se cumplan los requisitos y asunciones estadísticos de ANOVA (homocedasticidad, etc.), se llevarán a cabo modelos lineales con la puntuación pos-test o la puntuación de cambio como variable de resultado (VD, outcome) y las puntuaciones pretest incluidas como covariable eliminando así el sesgo sistemático y reduciendo la varianza del error. El nivel de significación será definido como p ≤ 0,05.  El tamaño del efecto será calculado mediante la d de Cohen para determinar la significación clínica: las diferencias insignificantes, pequeñas, medianas y grandes se reflejarán en tamaños de efecto de <0,2, 0,2-0,5, 0,5-0,8 y >0,8, respectivamente.  Para el análisis de la información cualitativa obtenida en los grupos focales se analizará mediante la metodología de análisis de contenido. Las sesiones se transcribirán literalmente en su totalidad por un solo investigador a partir de las grabaciones realizadas durante los grupos focales. Se realizará inicialmente una o varias lecturas completas hasta obtener una idea global de la información registrada y conseguir una inmersión en el texto. Posteriormente, se realizará una segunda lectura palabra a palabra, en la que se procederá a una codificación inductiva de las transcripciones, con el fin de capturar los conceptos y pensamientos clave, este proceso se realizarán por 2 investigadores de forma independiente. Posteriormente, los códigos se agruparán por su relación y vínculo en categorías. Dependiendo de las relaciones entre las subcategorías, los investigadores pueden comparar, combinar u organizar este mayor número de subcategorías en un número menor de categorías.  Se realizará una triangulación de investigadores, así como una triangulación metodológica (cuantitativa- cualitativa) para mejorar la calidad y la fiabilidad de los datos. Dos autores realizarán un contraste y comparación de códigos, subcategorías y categorías, refinando y redefiniendo cada uno de ellos para resolver posibles desacuerdos. Posteriormente, los hallazgos se enviarán al resto del equipo de estudio para ser comparados y debatidos, con el fin de alcanzar un consenso entre los investigadores.  ***Responsabilidad civil***  Se dispone de un seguro de responsabilidad civil específico para proyectos de investigación de la Universidad San Jorge que cubre a los participantes (Zúrich, número de póliza 86137890).  ***Consideración de la perspectiva de género***  Se tendrá en cuenta la inclusión equitativa entre los diferentes sexos durante el reclutamiento hasta obtener una prevalencia puntual por sexo similar a los últimos datos disponibles del sector sanitario o de población general en la ciudad de Zaragoza o la CCAA de Aragón(50). El trastorno depresivo mayor es una enfermedad con mayor prevalencia en el sexo femenino(51), por lo que se tendrá en cuenta si hay diferencias según sexo para poder ofrecer soluciones específicas.  ***Limitaciones***  La principal limitación será la previsible pérdida de pacientes que puedan completar el programa de fisioterapia expuesto, lo que puede ocasionar alargar en el tiempo las intervenciones para poder alcanzar el tamaño muestral. Otra limitación será la dificultad de controlar la cantidad de ejercicio físico realizado fuera de la intervención. La diversidad de subtipos de trastorno depresivo también podrá influir en los resultados, por lo que se tendrá en cuenta el diagnóstico concreto para minimizar este riesgo. También el estilo de vida previo al ingreso, si han tenido una vida activa o no podría influir en los resultados. Por otro lado, la muestra recibe el tratamiento estandarizado para su enfermedad, por lo que atribuir mejoras de forma exclusiva al ejercicio físico terapéutico y al plan de educación sanitaria será difícil de demostrar.  ***Memoria económica y fuente de financiación***  El proyecto “Efectividad de un programa de fisioterapia basado en ejercicio físico terapéutico y educación sanitaria dirigidos a mejorar la calidad de vida y el estado de salud en trastorno depresivo mayor: estudio de diseño mixto” cuenta con una financiación de 5700€ (ANEXO IV) otorgada a través de la Convocatoria Interna de Proyectos 2024-2025 de la Universidad San Jorge (ANEXO V).  El objeto de financiación a través del cual se valoró positivamente dicho proyecto fue la dedicación de presupuesto obtenido para:   - 2 publicaciones científicas en acceso abierto.   Siendo este uno de los costes elegibles de los proyectos presentados a dicha convocatoria, y con fecha límite de depósito de resultados en la entidad encargada de la publicación o registro a fecha fin de proyecto de 31 de diciembre de 2025.  En cuanto al uso de recursos necesarios para la puesta en marcha del proyecto de investigación, se adjunta en el ANEXO II la autorización por parte del responsable del Servicio de Psiquiatría del Sector I de Zaragoza y del director Médico del Hospital Universitario Royo Villanova para el uso del siguiente material dentro de las instalaciones del Hospital Universitario Royo Villanova de Zaragoza:   - Uso de la sala de audiovisuales del servicio de corta estancia de Psiquiatría del Hospital Universitario Royo Villanova de Zaragoza para las valoraciones, intervenciones y grupos focales del presente estudio. - Uso de las sillas y esterillas ya presentes en la misma sala de audiovisuales. - El coste asociado a este uso sería el propio destinado a la climatización e iluminación de la estancia durante el tiempo preciso para llevar a cabo el presente estudio.   También se incluyen con respectiva autorización (ANEXO I) de colaboración del personal laboral del Hospital Universitario Royo Villanova presente en este proyecto de investigación. |

| **4.7 Aspectos éticos** (balance riesgo/beneficio, **justificación en caso de solicitar exención del consentimiento** informado, implicaciones asistenciales, implicaciones para el participante o su familia, compensación a los participantes, póliza de seguro). |
| --- |
| Este estudio respeta los principios fundamentales establecidos en la Declaración de Helsinki, en el Convenio del Consejo de Europa relativo a derechos humanos y biomedicina, en la Declaración Universal de la UNESCO y la ley de protección de los datos personales de los participantes: Reglamento UE 679/2016 de protección de datos personales RGPD y Ley Orgánica 3/2018 de 5 de diciembre de Protección de Datos Personales y garantía de los derechos digitales LOPDGDD). Se mantendrá el anonimato y confidencialidad de todos los datos personales.  La participación será libre y voluntaria. Ningún participante recibirá compensación económica ni de ninguna otra índole por su participación. Se facilitará a cada posible participante la hoja de información al participante y en caso de aceptación, el documento de consentimiento informado, tanto a pacientes como a profesionales.  Una persona ajena a la evaluación y a la intervención realizará el proceso de anonimización de participantes. El documento de protección de datos y de consentimiento informado serán tenidos en cuenta. Antes de comenzar el estudio, se dispondrá de valoración positiva de los correspondientes comités de ética, así como de todos los convenios y autorizaciones pertinentes.  Todos los datos serán recogidos en tablas Excel protegidas con contraseña y ubicadas en Microsoft 365 con licencia de la USJ y anonimizadas. La IP creará una carpeta para compartir con el equipo investigador mediante la invitación específica a su correo @USJ. Sólo tendrán acceso los investigadores involucrados. Será una carpeta compartida con contraseña.  La transcripción de las grabaciones de audio se desarrollará en los quince días naturales tras la realización de los grupos focales. Una vez obtenidas las transcripciones las grabaciones serán borradas. Además, para estas grabaciones se utilizarán dispositivos de grabación de la Universidad San Jorge, bajo sistema de préstamo y se devolverán las mismas sin ningún tipo de material de audio. La finalidad de las grabaciones es la transcripción de los datos.  Para garantizar la seudonimización de los participantes en los grupos de discusión, el investigador que haga las transcripciones no estará presente en los grupos de discusión. Cada persona se identificará con un número y al comenzar la sesión dirá, por ejemplo, “Soy la voz 1” y así sucesivamente. Igualmente, se solicitará que cada persona antes de participar en el grupo de discusión diga el número de voz que es. Así, se facilitarán las transcripciones y anonimato.  Para el almacenamiento de los datos personales del proyecto, se utilizará el servicio Microsoft OneDrive al que se accede exclusivamente mediante las credenciales de los investigadores de este proyecto. Se llevarán a cabo copias de seguridad periódicas en el espacio Microsoft OneDrive y solo podrá acceder a la carpeta contenedora de la base de datos los tres investigadores USJ del proyecto. Durante el transcurso del proyecto de investigación, se evitará la transmisión electrónica de los datos personales o análisis de los resultados del proyecto, que no sean estrictamente necesarias para el adecuado desarrollo del mismo. |
| **4.8 Cronograma y plan de trabajo**:   - Etapas de desarrollo, duración, fechas estimadas de inicio y fin (indicar al menos mes y año). - Lugares donde se prevé realizar el proyecto, instalaciones que se utilizarán. |
| \| **Tareas** \| \| --- \| \| Mes  1 \| Mes 2 \| Mes 3 \| Mes 4 \| Mes 5 \| Mes 6 \| Mes 7 \| Mes 8 \| Mes 9 \| Mes 10 \| Mes 11 \| Mes 12 \| \| Aceptación comité de ética y convenios \| **x** \|  \|  \|  \|  \|  \|  \|  \|  \|  \|  \|  \| \| Reclutamiento \|  \| **x** \| **x** \| **x** \| **x** \| **x** \| **x** \| **x** \| **x** \| **x** \| **x** \|  \| \| Evaluación inicial \|  \| **x** \| **x** \| **x** \| **x** \| **x** \| **x** \| **x** \| **x** \| **x** \| **x** \|  \| \| Intervención \|  \| **x** \| **x** \| **x** \| **x** \| **x** \| **x** \| **x** \| **x** \| **x** \| **x** \|  \| \| Evaluación final \|  \|  \| **x** \| **x** \| **x** \| **x** \| **x** \| **x** \| **x** \| **x** \| **x** \|  \| \| Análisis de datos \|  \|  \|  \|  \|  \|  \| **x** \| **x** \| **x** \| **x** \| **x** \| **x** \| \| Publicaciones \|  \|  \|  \|  \|  \|  \|  \|  \|  \| **x** \| **x** \| **x** \|   Las instalaciones que se utilizarán son el aula de audiovisuales del servicio de corta estancia de psiquiatría del Hospital Universitario Royo Villanova.  Final de proyecto estimado en julio de 2026. |
