# Supplementary material for: Study protocol for a mixed-methods pilot of a physiotherapy plus education program for inpatients with major depressive disorder: Feasibility and preliminary effects
Source: PLoS One. 2025 Nov 6;20(11):e0326012. doi: 10.1371/journal.pone.0326012 (PMC12591423; doi:10.1371/journal.pone.0326012)
Supplement: S2 File — (DOCX) [file pone.0326012.s009.docx]

CEICA RESEARCH PROJECT REPORT

| **TITLE** | Effectiveness of a physical therapy program based on therapeutic physical exercise and health education aimed at improving quality of life and health status in major depressive disorder: a mixed design study. |
| --- | --- |
| **VERSION AND DATE** | Version 2, May-2025 |
| **CENTER WHERE THE STUDY IS PERFORMED** | Royo Villanova University Hospital (Zaragoza) |

| **PRINCIPAL INVESTIGATOR OF THE PROJECT** | |
| --- | --- |
| **NAME AND SURNAME** | José Lesmes Poveda López |
| **ID CARD** |  |
| **E-MAIL* E-MAIL* E-MAIL* E-MAIL* E-MAIL* E-MAIL** | jlpoveda@usj.es |
| **TELEPHONE*.** |  |
| **WORKSTATION** | Teaching and research staff |
| **SERVICE/DEPARTMENT** | Degree in Physiotherapy |
| **CENTER/ FACULTY-UNIVERSITY/ OTHER** | San Jorge University |

| **PROMOTER (Essential for clinical trials and observational drug studies)** | |
| --- | --- |
| **COMPANY IDENTIFICATION/ FIRST NAME AND SURNAME*.** |  |
| **TAX ID** |  |
| **E-MAIL* E-MAIL* E-MAIL* E-MAIL* E-MAIL* E-MAIL** |  |
| **TELEPHONE*.** |  |

| **contact of the person in charge of handling the request**  *If you wish, please add the contact details of the person in charge of handling the details of the request.* | |
| --- | --- |
| **NAME AND SURNAME** | José Lesmes Poveda López |
| **E-MAIL** | jlpoveda@usj.es |
| **TELEPHONE** |  |

The personal data that may be contained in this communication will be incorporated into the processing system for which the Aragonese Institute of Health Sciences (IACS) is responsible. The data will be processed for the management and follow-up of the studies evaluated by CEICA. The data will be deleted when the management and/or processing of the request has been answered and is no longer necessary. You have the right to access, rectify and delete the data, as well as the other rights granted by the data protection regulations before the IACS, domiciled at the Centro de Investigación Biomédica de Aragón. Avda. San Juan Bosco, nº 13, 500009, Zaragoza or by sending an e-mail to protecciondedatos.iacs@aragon.es.

**GLOSSARY**

- Anonymization: process by which it is no longer possible to establish by reasonable means the link between a piece of data and the subject to which it refers. It is also applicable to the biological sample.
- BIGAN: healthcare big data platform (managed by IACS) that allows access to Aragonese Health System data in a pseudonymized way for its use in management and research.
- Biobank: public or private, not-for-profit establishment that houses one or more collections of biological samples of human origin for biomedical research purposes, organized as a technical unit with criteria of quality, order and destination.
- Center: Institution where a study is conducted (hospital, health center, residence, faculty, private clinic, school, etc.). **In case of doubt, reference should be made to the place where the participants come from** (hospital, school, sports club, etc.).
- Confidentiality Commitment: Document that, on a mandatory basis, must be signed by students and residents who carry out any activity in the public health system (model established in Order SSI/81/2017).
- Informed consent: manifestation of free and conscious will validly issued by a capable person, or by his/her authorized representative, preceded by adequate information.
- CEICA opinion: document that certifies that the CEICA has evaluated a research project and that the project complies with the applicable legal norms and ethical criteria.
- Survey: collection of information in physical or digital support, with or without direct interaction with the source subject.
- Interview: collection of information with direct interaction with the source subject, through verbal responses.
- Research team: A group of researchers who jointly carry out a specific project.
- Primary data source: When data are collected directly from the study participant and for the purpose of the study.
- Secondary data source: When data already collected (and therefore recorded) that were obtained for a purpose other than the study (welfare, teaching, etc.) are used for the study.
- Research group: A group of researchers with a common trajectory (publications, financing) directed or coordinated by a Principal Investigator, grouped around a research topic and not necessarily linked to a health care or departmental affiliation.
- Participant information sheet: Document informing potential participants of the nature of the study, so that they can give their informed consent.
- Intervention (intervention study): any action to be performed on a person due to his/her participation in a study (it can be a pharmacological, physiotherapeutic treatment, an educational, behavioral, psychological intervention).
- Principal Investigator: Investigator who leads the project and is responsible for its design, conduct and dissemination of the results. If the study is multicenter, there should be a principal investigator in each center who is responsible for the patients, data and/or samples
- Biological sample: any biological material of human origin that can be preserved and that may contain information on the characteristic genetic endowment of a person.
- Routine clinical practice: Procedures that are carried out for purely health care purposes, regardless of whether or not an individual participates in a research study.
- Research project: scientific procedure aimed at gathering information and formulating hypotheses on a given social or scientific phenomenon.
- Promoter: individual, company, institution or organization responsible for initiating, managing, organizing and financing a study.
- Pseudonymization: the processing of personal data in such a way that they can no longer be attributed to a data subject without the use of additional information, provided that such additional information is separately identified and subject to technical and organizational measures designed to ensure that the personal data are not attributed to an identified or identifiable natural person.

| 1. **PROJECT SCOPE AND FINANCING** | |
| --- | --- |
| Is it a multicenter project? Yes No | If so, the complete list of centers must be submitted and a research team commitment must be filled out **for each center in Aragón** (see [Annex I](#A3)). |
| Do you have specific funding for the study? Yes No | In **all cases,** [Annex II](#A4): Authorization for the use of resources must be completed. In addition, if yes, the budget and source of financing must be submitted. |

| 1. **CHARACTERISTICS OF THE STUDY** | |
| --- | --- |
| **2.1 Is this research involving drugs**? Yes No | |
| If yes, please select one option:   1. This is an observational study on drug treatment (EOM).   In this case, specify:  Prospective Data Collection Retrospective Data Collection  Cross-sectional Collection   1. This is an intervention study: clinical trial with drugs.   In this case it must be submitted according to AEMPS instructions [(](https://www.aemps.gob.es/medicamentos-de-uso-humano/investigacionclinica_medicamentos/ensayosclinicos/#n-espanola)https://www.aemps.gob.es/medicamentos-de-uso-humano/investigacionclinica_medicamentos/ensayosclinicos/#n-espanola). | |
| **2.2 Is this research involving medical devices or medical devices**? Yes No | |
| If yes, please select one option:   1. This is an observational study on the use of the medical device. 2. This is an intervention study: clinical trial with medical devices.   In this case, it must be submitted according to CEICA's SOP for this type of studies [(](https://www.iacs.es/investigacion/comite-de-etica-de-la-investigacion-de-aragon-ceica/)https://www.iacs.es/investigacion/comite-de-etica-de-la-investigacion-de-aragon-ceica/). | |
| **2.3 Is this research involving invasive procedures?**  (Definition: any intervention performed for research purposes that involves a physical or psychological risk to the participant).  If so, an insurance policy must be taken out or a minimum risk must be justified. | Yes No |
| **2.4 Does the research involve minors or persons incapable of giving consent?**  If yes, an information and informed consent document addressed to the guardian/legal representative/family member and another addressed to the minor (adapted to his/her capacity) must be submitted. [Review CEICA template](https://www.iacs.es/wp-content/uploads/2022/12/Doc-1-23-Anexo-Plantilla-HIP-modif-2.doc). | Yes No |

| 1. **CHARACTERISTICS OF THE STUDY** | |
| --- | --- |
| **2.5 Are biological samples used in the study?** | Yes No |
| If yes, please select one or more options:   1. Samples of surplus care are used with consent for the project.   The information and consent document ([CEICA template](https://www.iacs.es/wp-content/uploads/2022/12/Doc-1-23-Anexo-Plantilla-HIP-modif-2.doc)) must be submitted.   1. Samples of surplus care samples used without consent   It must be adequately justified in the ethical aspects section (art. 58.2 Law 14/2007).   1. Samples are collected specifically for this study   The information and consent document ([CEICA template](https://www.iacs.es/wp-content/uploads/2022/12/Doc-1-23-Anexo-Plantilla-HIP-modif-2.doc)) must be submitted.   1. A private sample collection is created   For the creation of a new collection, submit the necessary documentation for evaluation and indicate the registration number (No. _____________) (see [CEICA website](https://www.iacs.es/investigacion/comite-de-etica-de-la-investigacion-de-aragon-ceica/ceica-evaluaciones-y-otras-presentaciones/ceica-proyectos-de-investigacion/)).   1. Samples already collected in a private sample collection are used.   In this case, identify the collection number and the person in charge: _____________________   1. Samples are requested from an authorized Biobank. In this case, identify the Biobank: _________   The application must be submitted to the | |
| **2.6 Is genetic analysis performed?** | Yes No |
| **2.7 Are embryos, human embryonic cells, human fetal cells or tissues or human pluripotent cells obtained by cell reprogramming used?**  You should contact IACS or the responsible institution for further approvals. | Yes No |

| 1. **PROCESSING OF PERSONAL DATA**   **Personal data** is considered to be any data (age, sex) or any information (numerical, alphabetical, graphic, acoustic) on an identified or identifiable natural person; any person whose identity can be determined, directly or indirectly (i.e. who has not been irreversibly anonymized at source) is considered to be identifiable. | |  |
| --- | --- | --- |
| **3.1 Is personal data collected or processed in the study?** | Yes No |  |
| If yes, check the applicable option:  The informed consent of the person concerned is requested  Submit the information and consent document ([CEICA template](https://www.iacs.es/wp-content/uploads/2022/12/Doc-1-23-Anexo-Plantilla-HIP-modif-2.doc)).  Data obtained for another purpose that have been pseudonymized (e.g., medical history, other research, other records) are used in accordance with A.D. 17 of Organic Law 3/2018.  Other. Specify: | |  |
| Check the **categories** to which the collected data belong:  **identification data** (name, address, email, DNI, medical record number, telephone, signature, IP, geolocation, image/voice, other)  **personal data:** date of birth, place of birth, parents' names, place of work, economic data, sex, marital status, children, academic qualifications, other.  **opinion data**  **particularly sensitive data**: health, ethnicity, religion, political opinion, sexual life or orientation, union membership, special educational needs, etc. | |  |
| - 1. **If the data is collected directly from the data subject (primary source), specify the procedure** (Example: interview, paper survey, email, telephone, web applications, ....   Personal interview with the interested party and on-site evaluation by research team personnel.  Focus groups will be carried out in a semi-structured manner, in which a series of open questions will be proposed through which qualitative data will be obtained for subsequent analysis. These focus groups will be carried out in person, and will be recorded using an audio for subsequent transcription and analysis. The use of the recording will be solely and exclusively for the analysis of the study. The audio recordings collected will be manually transcribed into a Microsoft Word document to be read and analyzed. All the information will be deposited in a Microsoft Sharepoint application licensed by the Universidad San Jorge to which only the research team will have access. The Information Sheet and Informed Consent documents for participants will be delivered and collected by the principal investigator and in collaboration with the external collaborating researchers Ms. Ana Villagrasa Cantín and Ms. Sara Pérez Mansilla, and the documents will be kept during the study by the principal investigator at the facilities of the Universidad San Jorge.  The same procedure will be followed for the collection of personal data from the professionals participating in their specific focus groups. This data collection will be done from the primary source directly through an interview prior to the start of the focus group.  **3.3 If not collected directly from the data subject (secondary source),** check the option and specify:  Data is collected from an existing record (e.g., medical history) **with the consent** of the data subject.  Data from other similar research, for which the consent of the interested party was obtained, are reused. Permission from the person responsible for the data, the consent form with which they were obtained and the commitment to use the pseudonymized data must be submitted ([see on the web](https://www.iacs.es/wp-content/uploads/2022/12/Doc-5.-declaracion-responsable-datos-seudonimizad.docx)).  Data obtained for another purpose and **without consent** are used **for research** (e.g., medical history or other record).  In this case, indicate:  The investigator (if the investigator is staff of the center) has direct access to the clinical history. Present permission from the person responsible for the data (if it is the clinical history, authorization from the management must be presented for access to data for this study).  The researcher receives the data already pseudonymized Present the commitment to use the pseudonymized data ([see on the web](https://www.iacs.es/wp-content/uploads/2022/12/Doc-5.-declaracion-responsable-datos-seudonimizad.docx)).  Note: **researchers who do not have an employment relationship with the hospital/center** do not have access to the medical records, so ALWAYS use this source to obtain pseudonymized data.  In all cases, explain: origin of the data, data controller.  Only the researcher Bárbara Marco Gómez, who is part of the hospital center, will have access to the clinical history. The data extracted from the clinical history will be date of birth, age, gender, profession, height, weight, BMI, medical diagnoses, pharmacological treatment and other non-pharmacological treatments, if any. The data will be obtained from the personal interview and specific evaluation of the interested party by the collaborating research staff of the short-stay psychiatry unit of the Royo Villanova University Hospital and transferred to the data collection notebook (CRD) in a pseudonymized form. The CRD will be kept in the office of the psychiatry service until immediate delivery to the principal investigator, who will keep them following the appropriate protective measures, in a locked locker at the Universidad San Jorge, then transferring the information to an Excel format in the shared Microsoft 365 Sharepoint folder licensed by the Universidad San Jorge with the researcher Juan Francisco Roy Delgado, and thereafter they can be consulted by the rest of the research team.  Data from BIGAN  Submit a report from the Biocomputing Unit ([link to the application](https://www.iacs.es/instituto-aragones-ciencias-la-salud/oficina-virtual/solicitud-de-acceso-a-datos-para-realizacion-de-un-proyecto-de-investigacion-rpi01-3a/)) and a commitment to use pseudonymized data ([see on the website](https://www.iacs.es/wp-content/uploads/2022/12/Doc-5.-declaracion-responsable-datos-seudonimizad.docx)). | |  |
| **3.4 Once the information and data have been obtained, how is the privacy of the participants guaranteed?**  Only aggregated data are used (i.e., data that correspond to groups of people and not to each individual).  The data is anonymized (the data cannot be associated with an identified or identifiable person because the link to any information that identifies the subject has been irreversibly destroyed  data are pseudonymized or coded (direct identifiers are replaced by a code/pseudonym known only to the research team)  Explain how and by whom the adopted measure is carried out:  Assignment of an alphanumeric identification code by the researcher Bárbara Marco Gómez. All paper documents will be kept in a locked locker at the Universidad San Jorge by the principal investigator, José Lesmes Poveda López. The pseudonymized data will be organized in an Excel workbook and an SPSS file, which will contain a password for access to both the file and the computer. | |  |
| **3.5 Data retention period:** specify date of destruction (at least month and year)  July 2026. | |  |
| **3.6 Data Processors (do not fill in in case of anonymous data)**  A **data processor** is any natural or legal person, public authority, service or other body that processes personal data, external to the controller (researcher).  If a third party (outside the institution) processes project data, it will be necessary to sign a **processor contract**. A model can be downloaded from <https://seguridad.salud.aragon.es/plantillas/>  Indicate which persons will process the data collected, specifying who will have access to the identifying data:  The researcher Bárbara Marco Gómez will be in charge of data collection and pseudonymization treatment. The rest of the pseudonymized data processing tasks will be performed by José Lesmes Poveda López and Juan Francisco Roy Delgado. The rest of the research team will have access to the data.  Are all persons authorized to process the data subject to a confidentiality agreement signed with the center?  Yes No | | |
| **3.7 Will data be disclosed to third parties?** Yes No  In case of assignment, it must be specified:  - the data provided are: identified, pseudonymised, anonymised  - to whom they are assigned  - what data is transferred  - for what purpose  - Explain how data are pseudonymized or anonymized:  - If there are international transfers: specify company and country (in this case, the express consent of the participant must be requested for this transfer). | | |

| **PROCESSING OF PERSONAL DATA** | |
| --- | --- |
| **3.8 Are recordings (audio/video) to be made?**  Remember that the express consent of the data subject is required to make recordings, this information must be included in the consent document. | Yes No |
| In case of recording, it must be specified:  Express consent to audio recording of the focus groups will be included in the informed consent forms. Likewise, at the beginning of the focus groups, a reminder will be given that they will be recorded. Participants may object to such recording at any time. The content of the focus groups will be recorded on an audio recorder  - Where they are to be kept, who has access and the security measures to be applied:  The audio files extracted from the recordings will be kept in a Microsoft 365 Sharepoint folder licensed by the Universidad San Jorge, which will only be accessible from corporate accounts with passwords. Access will also only be possible from the researchers' computers protected with password access. All project researchers will have access to the data.  - Time period for preservation of the recordings  After transcription of the recordings, within 15 days after each recording.  - for what purpose:  The purpose of obtaining and analyzing the data extracted from the audio recording will be solely for the research purposes indicated in this report. The purpose of the recording will be the transcription of this information later to be used for further analysis.  - if computer applications or cloud storage are used, who the service provider is and where it is legally resident, as well as the link to its privacy policy, should be indicated:  Microsoft 365 license owned by St. George University, Atlas-Ti 24: ATLAS.ti Scientific Software Development GmbH. Bergmannstraße 68. D-10961 Berlin. Germany E-mail: [privacy@atlasti.com](mailto:privacy@atlasti.com)[dataprotection@atlasti.com](mailto:dataprotection@atlasti.com) .  <https://atlasti.com/es/legal/politica-de-privacidad> | |
| **3.9 Information security measures: description of the information systems to be used**  **It is reminded that servers containing personal data must be located in the territory of the EU (RDL 14/2019).** | |
| - System in which the data is to be stored (personal computer, corporate servers, external company or organization, cloud service provider, etc.)  The documents will be saved through the use of an Excel document located in Microsoft Sharepoint 365 license owned by Universidad San Jorge. The Excel file will be password protected, located in a folder created by the principal investigator and shared only with the members of the research team involved. This folder will also be password protected. And only the principal investigator and the researchers of the project will have access to this folder.  - Applications to be used for data processing (excel, spss, etc.)  Excel in Microsoft Sharepoint 365 Sharepoint license owned by Universidad San Jorge. The data will be processed in the computer software SPPS v.28, Atlas-Ti 24. All data will be password protected.  - If online computer applications or "cloud" storage is used, it should indicate who the service provider is and where their legal residence is located, as well as the link to their privacy policy.  The Microsoft 365 software package will be used, the supplier being Universidad San Jorge, Campus Universitario, Autovía Mudéjar, km. 299, 50830 Villanueva de Gállego, Zaragoza https://www.usj.es/politica-de-privacidad. | |
| **3.10 Information Security Measures: Devices** | |
| - Indicate whether any type of removable device (portable USB, external hard disk, etc.) is to be used and whether it is to be encrypted.  No removable device is to be used.  - If corporate computer systems are not used, indicate whether backups are made.  - Indicate the security measures for paper documents (custody, access).  Indicate the person who is the custodian of the informed consents and satisfaction surveys (paper) collected from patients and participating professionals.  The documents in paper format will be kept by the principal investigator, José Lesmes Poveda López, in the locked locker at the Universidad San Jorge in Villanueva de Gállego (Zaragoza). | |
| **General recommendations on data use**   - Do not use Wifi networks to transmit sensitive information. - Use strong passwords and change them periodically. - Always encrypt sensitive information to be sent by e-mail. - Ensure that the versions of operating systems and applications are always up to date. - On personal computers, always use antivirus software that is up to date. - Never open files attached to e-mails in which you do not identify the sender. - Do not use social networks to communicate sensitive information. - The cell phone is not a very secure device for handling sensitive information, and the antivirus programs that can be installed offer little protection. - Use corporate applications whenever possible - The use of USB or other removable devices is strongly discouraged. | |

**4. DESCRIPTION OF RESEARCH PROJECT (complete fields or attach complete protocol with equivalent information)**

| **4.1 Tasks of the research team**  Briefly explain who is participating in the study, in what capacity and what tasks they will perform, as well as their affiliation (current job title). The cv and signature of all of them must be submitted in [Annex I.](#A3)  If the study is multicenter, an Annex I must be submitted for each center. |
| --- |
| - Mr. José Lesmes Poveda López. Physiotherapist. Teacher and researcher at the Universidad San Jorge (Zaragoza). Faculty of Health Sciences. iPhysio Research Group. Responsible Researcher of the project - Dr. Carolina Jiménez Sánchez. Physiotherapist. Teacher and researcher at San Jorge University (Zaragoza). Faculty of Health Sciences. iPhysio Research Group. - Ms. Raquel Lafuente Ureta. Physiotherapist. Teacher and researcher at San Jorge University (Zaragoza). Faculty of Health Sciences. iPhysio Research Group - Dr. Marta Guarch Rubio. Psychologist. Teacher and researcher at San Jorge University (Zaragoza). Faculty of Health Sciences. IIPOV Research Group. - Dr. Juan Francisco Roy Delgado. Psychologist. Teacher and researcher at San Jorge University (Zaragoza). Faculty of Health Sciences. IIPOV Research Group - Dr. Barbara Marco-Gomez. Psychiatrist. Psychiatry Short Stay Unit of the HURV. Zaragoza. - Mrs. Ana Villagrasa Cantín. Nursing Supervisor. HURV Psychiatry Short Stay Unit. Zaragoza - Mrs. Sara Pérez Mansilla. Nurse specialist in Mental Health. Unit of short stay of Psychiatry of the HURV. Zaragoza.   The tasks of each researcher are detailed below:   \|  \| José Lesmes Poveda López \| Carolina Jiménez Sánchez \| Raquel Lafuente Ureta \| Marta Guarch Rubio \| Juan Francisco Roy Delgado \| Barbara Marco Gomez \| Ana Villagrasa Cantín \| Sara Pérez Mansilla \| \| --- \| --- \| --- \| --- \| --- \| --- \| --- \| --- \| --- \| \| Study design \| **x** \| **x** \| **x** \| **x** \| **x** \| **x** \|  \|  \| \| Data processing \| **x** \|  \| **x** \|  \| **x** \| **x** \|  \|  \| \| Selection of participants \|  \|  \|  \|  \|  \| **x** \| **x** \| **x** \| \| Access to data \| **x** \| **x** \| **x** \| **x** \| **x** \| **x** \| **x** \| **x** \| \| Pre-intervention evaluation \| **x** \|  \|  \|  \|  \| **x** \| **x** \| **x** \| \| Post-intervention evaluation \| **x** \|  \|  \|  \|  \| **x** \| **x** \| **x** \| \| Intervention \| **x** \|  \|  \|  \|  \|  \|  \|  \| \| Qualitative evaluation \|  \| **x** \| **x** \|  \|  \| **x** \| **x** \| **x** \| \| Analysis of results \|  \| **x** \| **x** \| **x** \| **x** \|  \|  \|  \| \| Interpretation of results \|  \| **x** \| **x** \| **x** \| **x** \|  \|  \|  \| \| Elaboration and dissemination of research results \| **x** \| **x** \| **x** \| **x** \| **x** \| **x** \| **x** \| **x** \| |
| **4.2 Justification of the study: Background, current state of the subject, relevance** (Cite bibliographic references in the following section). |
| The World Health Organization(1) indicates that mental health is the state of health that allows people to face stressful moments with capacities and abilities, which is a fundamental human right and a necessary element for community development. Its decline leads to mental illness, characterized by situations of distress, functional disability, greater difficulty in basic and instrumental activities of daily living, sedentary lifestyle and muscle atrophy, poor quality of life, low muscle strength and fatigue, pain, changes in muscle tone, cognitive and affective deterioration, social isolation, stigmatization, job loss and risk of self-harm and premature death, in addition to generating an overload of care in public health services and increased costs .(2)  The prevalence of mental health problems in Spain reaches 27.4% in the global population, being major depressive disorder the mental illness with the highest incidence rate in the adult population (4.1% of the Spanish adult population and 5% in the adult population worldwide), followed by anxiety disorders, personality disorders, psychotic disorders, cognitive disorders, anorexia nervosa, bulimia and obsessive-compulsive disorders(3) . After the first year following the Covid-19 pandemic, mental illnesses increased in prevalence by 25% worldwide, mainly depressive and anxiety disorders .(4)  Major depressive disorder, so classified according to the international classification of diseases in its tenth version (ICD-10)(5) and by the manual of mental disorders DSM-5 of the American Psychiatric Association. (6)is a mood disorder, characterized by a deep sadness and loss of interest in any activity in a sustained manner, for at least two weeks, involving a change in the baseline state of the person, not explainable by another disease, by the consumption of toxic or pharmacological. It is a disease that is associated with a very important loss of quality of life, accompanied by both psychological symptoms (anhedonia, anergy, sleep and appetite disturbance) and physical symptoms due to functional impairment, thus being the leading cause of disability worldwide(7) , affecting more women(8) and the population with lower income(9,10) , causing severe suffering and alteration on work, family and social activities, and that as a major consequence would be suicide(11,12) . It is a disease that can be a single episode or recurrent for more than two years, with a severity of mild, moderate or severe(13) . All these problems significantly affect the population, which requires a comprehensive approach for its management.  We must also take into account the aging process of the population, currently 19% of the population over 65 years of age in Spain, and with a forecast of more than 25% in 10 years(14) . In this population, the state of poor mental health causes a higher risk of comorbidity and disability rate(15) , loneliness, stigmatization and institutionalization(16,17) , which affects the increased risk of mortality(18,19) . The conditioning factors of aging mean that this population requires more exhaustive follow-up due to the severity of its repercussions and the increased expenditure and use of healthcare resources(20)  Thus, there should be a program of promotion, intervention and education for health in this group of people with major depressive disorder to favor their recovery, under an interdisciplinary coordination, being necessary the training and promotion of specialized health professionals who can improve this situation(8,21,22) . The WHO considers the promotion of mental health to be of great interest and urges organizations and states to implement action plans based on three transformation paths: to give more value to mental health, to act on the physical, social and economic condition, and finally to strengthen a community network of support services .(1)  Major depressive disorder is a multifactorial illness, influenced by genetic and environmental factors(23) . The presence of these factors leads to a decrease in the neurotransmitters serotonin, norepinephrine and dopamine. This dysfunction is mediated by hyperactivity of the hypothalamic-pituitary-adrenal (HHA) axis responsible for stress control and the presence of cortisol, and a decrease in the morphological volume of the hippocampus due to a decrease in dendrites and neuronal networks(24,25) . Major depressive disorder is considered an inflammatory disease due to the increase of proinflammatory cytokines in blood in these patients, caused by this activation of the HHA axis . (7)  The usual treatments for major depressive disorder are interdisciplinary in approach, including mainly the medical pillar(26–28) (primary and specialized care), pharmacological(29–31) , psychotherapeutic(32,33) , and to a lesser extent, physiotherapy(34,35) . The gateway to the care system is in primary care, where the medical professional makes the first clinical diagnosis and makes decisions on the necessary pharmacological treatment, assesses the level of severity of symptoms through screening tools and evaluation scales, in order to have the information for follow-up and to evaluate the need to move on to another level of care. The support of the psychiatrist will be necessary in the long-term follow-up of patients when the first line of action is not effective, and to determine the referral to psychology, occupational therapy or physiotherapy professionals. Important is the role of mental health nursing, which in addition to being a recognized specialty in the care of these patients, serves as a follow-up and analysis of the disease process in support of medicine. In general, pharmacology can have an acceptable good result, but the lack of adherence and control over its use is not always adequate, and in some cases the adverse effects they cause lead the patient to a state of drowsiness that hinders their independence(36) . As for psychotherapy, cognitive-behavioral therapy techniques, problem-solving or life review, among others, are common to treat mainly the affective and emotional symptoms most affected in major depressive disorder .(32)  The World Confederation for Physical Therapy (WCPT) defines physical therapy as a service it provides to people to develop, maintain and restore maximum movement and functional ability throughout life(37) . This care is provided in circumstances where movement and function are affected by aging, injury, pain or disease, with the understanding that movement is fundamental to health. Physiotherapists are concerned with identifying the level of quality of life of people and propose a treatment through movement that promotes, prevents, intervenes and rehabilitates their capabilities impaired by the disease, which not only covers the physical sphere, but also the psychological, emotional and social, emphasizing the necessary addition of health education(38) .It is the competence of physiotherapy to remain aligned with the needs of the population, and to investigate and attend to those health guidelines that are key to the development of the health of its environment, always in strict compliance with its code of ethical principles. Physiotherapy is an independent health profession, but it needs to be integrated into an interdisciplinary team in order to detect, plan and ensure all the factors that predispose to the improvement of the mental health of patients. Its functions include :(39)   - Conduct interviews with mental health professionals and patients to learn about treatment needs, goals and expectations. - Perform general health assessments, and specific physiotherapy assessments of musculoskeletal, respiratory and circulatory capacities. - To design a strategic treatment plan according to the needs of the service and the type of stay in the psychiatric ward, promoting group work among several patients, and if necessary, with individual interventions. - To detect the risk factors that may alter the health status of the patient in psychiatry service, which decrease the quality of life and life expectancy. - To carry out a strategic health education plan, seeking to promote healthy habits that favor patients' mood and strengths. - Conduct periodic reviews of patients' health status. - Conduct interdisciplinary team meetings.   To evaluate the effectiveness of the treatment in all its dimensions, both on the physical and mental pathology through the investigation of techniques and procedures based on science, with the purpose of being able to establish a clinical reasoning with evidence that allows to defend the need of the physiotherapeutic treatment.  Physiotherapy in mental health, and specifically for the patient with major depressive disorder, allows to work comprehensively on the patient, improving the mind and body connection through movement, making the person more aware of their capabilities and needs, giving tools for strengthening and confronting the difficulties they may encounter in performing tasks in their daily lives(40) . Many physical symptoms that may arise from major depressive disorder can be treated to empower the patient and allow them to be more active in overcoming stressful events, in addition to the fact that movement can improve mood through the release of endorphins(41) . The treatment of chronic pain associated with states of depression has had clinically significant effects(42–44) , which in turn have had an impact on the functional improvement of the affected persons, thus, the performance of therapeutic exercise through physiotherapy ensures a professional and aligned treatment with the rest of the health care providers that will improve the emotional and physical state(35).  ***Project justification***  Due to the high prevalence of major depressive disorder, its severe impact on the health of the adult patient, how it affects the community and the healthcare system, it would be pertinent to explore physiotherapy treatments of therapeutic physical exercise and health education and evaluate their effectiveness on their quality of life and health status, as a complement to the psychiatric and/or psychological treatments already present, in the setting of a short-stay inpatient psychiatric unit. |
| **4.3 Bibliography** (must be referenced in the preceding text) |
| 1. World Mental Health Report Transforming Mental Health for All. Overview. Geneva: World Health Organization; 2022.  2. Model of care for people with severe mental illness. Madrid: Instituto de Mayores y Servicios Sociales; 2007.  3. Report Salud mental en datos: prevalencia de los problemas de salud y consumo de psicofármacos y fármacos relacionados a partir de los registros clínicos de atención primaria (Mental health in data: prevalence of health problems and consumption of psychotropic and related drugs from primary care clinical records). Spanish Ministry of Health; 2020.  4. Santomauro DF, Mantilla Herrera AM, Shadid J, Zheng P, Ashbaugh C, Pigott DM, et al. Global prevalence and burden of depressive and anxiety disorders in 204 countries and territories in 2020 due to the COVID-19 pandemic. The Lancet. November 2021;398(10312):1700-12.  5. ICD-10-EN: International Classification of Diseases - 10.Œ revision : clinical modification. 2nd ed. January 2018. Madrid: Ministry of Health, Social Services and Equality; 2018.  6. Diagnostic and statistical manual of mental disorders: DSM-5. 5.^a^ed., 2.^a^reimp. Buenos Aires [etc.]: Editorial Médica Panamericana; 2016.  7. Beurel E, Toups M, Nemeroff CB. The Bidirectional Relationship of Depression and Inflammation: Double Trouble. Neuron. July 2020;107(2):234-56.  8. Dotson VM, Hsu FC, Langaee TY, McDonough CW, King AC, Cohen RA, et al. Genetic Moderators of the Impact of Physical Activity on Depressive Symptoms. J Frailty Aging. 2016;5(1):6-14.  9. Maier A, Riedel-Heller SG, Pabst A, Luppa M. Risk factors and protective factors of depression in older people 65+. A systematic review. Bayer A, editor. PLoS ONE. May 13, 2021;16(5):e0251326.  10. Cleary JL, Fang Y, Zahodne LB, Bohnert ASB, Burmeister M, Sen S. Polygenic Risk and Social Support in Predicting Depression Under Stress. AJP. Feb 1, 2023;180(2):139-45.  11. Ribeiro JD, Huang X, Fox KR, Franklin JC. Depression and hopelessness as risk factors for suicide ideation, attempts and death: meta-analysis of longitudinal studies. Br J Psychiatry. May 2018;212(5):279-86.  12. O'Connor SJ, Hewitt N, Kuc J, Orsini LS. Predictors and Risk Factors of Treatment-Resistant Depression: A Systematic Review. J Clin Psychiatry [Internet]. Nov 13, 2023 [cited Aug 28, 2024];85(1). Available from: https://www.psychiatrist.com/jcp/predictors-risk-factors-treatment-resistant-depression-systematic-review/  13. Dávila Hernández A, González González R, Liangxiao M, Xin N. Synomedical study of the pathophysiology of depression. International Journal of Acupuncture. 2016 Jan;10(1):9-15.  14. Mayores a un clic - Instituto de Mayores y Servicios Sociales [Internet]. [cited 2024 Aug 28, 2024]. Available from: https://imserso.es/espacio-mayores/estadisticas/mayores-un-clic  15. Boehlen FH, Herzog W, Maatouk I, Saum KU, Brenner H, Wild B. Treatment preferences of elderly patients with mental disorders. Z Gerontol Geriatr. Feb 2016;49(2):120-5.  16. Boström G, Conradsson M, Hörnsten C, Rosendahl E, Lindelöf N, Holmberg H, et al. Effects of a high-intensity functional exercise program on depressive symptoms among people with dementia in residential care: a randomized controlled trial. Int J Geriatr Psychiatry. Aug 2016;31(8):868-78.  17. Conradsson M, Littbrand H, Lindelof N, Gustafson Y, Rosendahl E. Effects of a high-intensity functional exercise programme on depressive symptoms and psychological well-being among older people living in residential care facilities: A cluster-randomized controlled trial. Aging Ment Health. July 2010;14(5):565-76.  18. Zhang Z, Jackson SL, Gillespie C, Merritt R, Yang Q. Depressive Symptoms and Mortality Among US Adults. JAMA Netw Open. Oct 9, 2023;6(10):e2337011.  19. Von Below A, Hällström T, Sundh V, Björkelund C, Hange D. Association between anxiety and depression and all-cause mortality: a 50-year follow-up of the Population Study of Women in Gothenburg, Sweden. BMJ Open. November 2023;13(11):e075471.  20. Underwood M, Lamb S, Eldridge S, Sheehan B, Slowther A, Spencer A, et al. Exercise for depression in care home residents: a randomised controlled trial with cost-effectiveness analysis (OPERA). Health Technol Assess [Internet]. 2013 May [cited 2013 Apr 21, 2024];17(18). Available from: https://www.journalslibrary.nihr.ac.uk/hta/hta17180/  21. Price RB, Duman R. Neuroplasticity in cognitive and psychological mechanisms of depression: an integrative model. Mol Psychiatry. March 2020;25(3):530-43.  22. Fox ME, Lobo MK. The molecular and cellular mechanisms of depression: a focus on reward circuitry. Mol Psychiatry. december 2019;24(12):1798-815.  23. Pitsillou E, Bresnehan SM, Kagarakis EA, Wijoyo SJ, Liang J, Hung A, et al. The cellular and molecular basis of major depressive disorder: towards a unified model for understanding clinical depression. Mol Biol Rep. Jan 2020;47(1):753-70.  24. Hussenoeder FS, Jentzsch D, Matschinger H, Hinz A, Kilian R, Riedel-Heller SG, et al. Depression and quality of life in old age: a closer look. Eur J Ageing. March 2021;18(1):75-83.  25. Jung ES, Choi YY, Lee KH. Effects of Integrative Cognitive Function Improvement Program on Cognitive Function, Oral Health, and Mental Health in Older People: A Randomized Clinical Trial. International Journal of Environmental Research and Public Health [Internet]. 2022;19(21). Disponible en: https://www.scopus.com/inward/record.uri?eid=2-s2.0-85141552646&doi=10.3390%2fijerph192114339&partnerID=40&md5=ffbce39036c5a7ce3a8bbb858568c5ed  26. Sukhato K, Lotrakul M, Dellow A, Ittasakul P, Thakkinstian A, Anothaisintawee T. Efficacy of home-based non-pharmacological interventions for treating depression: a systematic review and network meta-analysis of randomised controlled trials. BMJ Open. July 2017;7(7):e014499.  27. Goodwin GM, Stein DJ. Generalised Anxiety Disorder and Depression: Contemporary Treatment Approaches. Adv Ther. September 2021;38(S2):45-51.  28. Baba H. Treatment strategy for late-life depression. PCN Reports. June 2023;2(2):e91.  29. Höppner J, Schulz M, Irmisch G, Mau R, Schläfke D, Richter J. Antidepressant efficacy of two different rTMS procedures. High frequency over left versus low frequency over right prefrontal cortex compared with sham stimulation. Eur Arch Psychiatry Clin Neurosci. April 2003;253(2):103-9.  30. Kishi T, Ikuta T, Sakuma K, Okuya M, Hatano M, Matsuda Y, et al. Antidepressants for the treatment of adults with major depressive disorder in the maintenance phase: a systematic review and network meta-analysis. Mol Psychiatry. Jan 2023;28(1):402-9.  31. Nunez NA, Joseph B, Pahwa M, Kumar R, Resendez MG, Prokop LJ, et al. Augmentation strategies for treatment resistant major depression: A systematic review and network meta-analysis. Journal of Affective Disorders. April 2022;302:385-400.  32. Cuijpers P, Karyotaki E, Eckshtain D, Ng MY, Corteselli KA, Noma H, et al. Psychotherapy for Depression Across Different Age Groups: A Systematic Review and Meta-analysis. JAMA Psychiatry. July 1, 2020;77(7):694.  33. Bhattacharya S, Kennedy M, Miguel C, Tröger A, Hofmann SG, Cuijpers P. Effect of psychotherapy for adult depression on self-esteem: A systematic review and meta-analysis. Journal of Affective Disorders. March 2023;325:572-81.  34. Lialy HE, Mohamed MA, AbdAllatif LA, Khalid M, Elhelbawy A. Effects of different physiotherapy modalities on insomnia and depression in perimenopausal, menopausal, and post-menopausal women: a systematic review. BMC Women's Health. July 8, 2023;23(1):363.  35. Noetel M, Sanders T, Gallardo-Gomez D, Taylor P, Del Pozo Cruz B, Van Den Hoek D, et al. Effect of exercise for depression: systematic review and network meta-analysis of randomised controlled trials. BMJ. February 14, 2024;e075847.  36. Hennessy S, Leonard C, Gagne J, Flory J, Han X, Brensinger C, et al. Pharmacoepidemiologic Methods for Studying the Health Effects of Drug-Drug Interactions. Clin Pharma and Therapeutics. 2016 Jan;99(1):92-100.  37. International Organization for Physical Therapy in Mental Health (IOPTMH) \| World Physical Therapy [Internet]. [cited 2024 Aug 28, 2024]. Available from: https://world.physio/es/subgroups/mental-health  38. Breitve MH, Hynninen MJ, Kvåle A. The effect of psychomotor physical therapy on subjective health complaints and psychological symptoms. Physiother Res Int. Dec 2010;15(4):212-21.  39. International Organization of Physical Therapy in Mental Health [Internet]. [cited 2024 Aug 28, 2024]. Available from: https://www.ioptmh.org/  40. Ayaz EY, Dincer B, Mete E, Benli RK, Cinbaz G, Karacan E, et al. Evaluating the impact of aerobic and resistance green exercises on the fitness, aerobic and intrinsic capacity of older individuals. Arch Gerontol Geriatr. March 2024;118:105281.  41. Schuch FB, Vancampfort D. Physical activity, exercise, and mental disorders: it is time to move on. Trends Psychiatry Psychother [Internet]. 2021 [cited 2023 Aug 25, 2023]; Available from: https://www.scielo.br/j/trends/a/jCRvs9LQq8ycmLwLysGBRFM/?lang=en  42. Fernández-Pérez P, Leirós-Rodríguez R, Marqués-Sánchez MP, Martínez-Fernández MC, De Carvalho FO, Maciel LYS. Effectiveness of physical therapy interventions in women with dyspareunia: a systematic review and meta-analysis. BMC Women's Health. July 24, 2023;23(1):387.  43. Kannan P, Lam HY, Ma TK, Lo CN, Mui TY, Tang WY. Efficacy of physical therapy interventions on quality of life and upper quadrant pain severity in women with post-mastectomy pain syndrome: a systematic review and meta-analysis. Qual Life Res. Apr 2022;31(4):951-73.  44. Cohen-Biton L, Buskila D, Nissanholtz-Gannot R. Review of Fibromyalgia (FM) Syndrome Treatments. IJERPH. Sep 24, 2022;19(19):12106.  45. Hernandez G, Garin O, Pardo Y, Vilagut G, Pont À, Suárez M, et al. Validity of the EQ-5D-5L and reference norms for the Spanish population. Qual Life Res. September 2018;27(9):2337-48.  46. Lobo A, Chamorro L, Luque A, Dal-Ré R, Badia X, Baró E. Validation of the Spanish versions of the Montgomery-Asberg Depression Rating Scale and the Hamilton Anxiety Rating Scale for the assessment of depression and anxiety. Medicina Clínica. January 2002;118(13):493-9.  47. Hjermstad MJ, Fayers PM, Haugen DF, Caraceni A, Hanks GW, Loge JH, et al. Studies Comparing Numerical Rating Scales, Verbal Rating Scales, and Visual Analogue Scales for Assessment of Pain Intensity in Adults: A Systematic Literature Review. Journal of Pain and Symptom Management. June 2011;41(6):1073-93.  48. Scholz U, Gutiérrez Doña B, Sud S, Schwarzer R. Is General Self-Efficacy a Universal Construct?1. European Journal of Psychological Assessment. September 2002;18(3):242-51.  49. Gómez Ramírez OJ, Carrillo González GM, Cárdenas DC. Survey of satisfaction with health care in people with chronic disease. eglobal. Sep 27, 2016;15(4):321.  50. Government of Aragon. Report on the prevalence of depression in Aragón 2022. Zaragoza: Gobierno de Aragón; 2022 .  51. Salk RH, Hyde JS, Abramson LY. Gender differences in depression in representative national samples: meta-analyses of diagnoses and symptoms. Psychological Bulletin. August 2017;143(8):783-822. |
| **4.4 Hypothesis** (assertion to be proved) |
| The intervention with a physical therapy program based on therapeutic physical exercise and health education, together with the usual treatments in adult patients with major depressive disorder, in a short-stay psychiatric unit, shows effectiveness comparing pre and post intervention values on quality of life and health status, measured through validated scales. |
| **4.5 Objectives** |
| ***Main objective***  To analyze the effectiveness of a physical therapy intervention program based on therapeutic physical exercise and health education on the general, mental and physical health status and quality of life of adults with major depressive disorder during their admission to a short-stay psychiatric unit of the Royo Villanova Hospital in Zaragoza, measured by means of the EQ-5D-3L quality of life scale .(45)  ***Secondary objectives***  To analyze the effectiveness on clinical depression symptoms, measured through the Montgomery-Asberg Depression Rating Scale in its Spanish validated version .(46)  To analyze the effectiveness of the pain symptom, measured through the NRS scale .(47)  To analyze the effectiveness on self-efficacy, measured through the General Self-Efficacy Scale .(48)  To analyze the level of satisfaction with the physical therapy intervention program, measured through the Satisfaction with Health Care Satisfaction Survey for People with Chronic Illness (GCPC-UN-ESU) .(49)  To describe through qualitative focus groups the experiences and perceptions of the participants with the disease and in the physical therapy intervention program, as well as the perceptions about the management of their process, and the barriers and facilitators that the treatments, including the one proposed in this project, pose for them.  To describe through qualitative focus groups the perceptions of the professionals who make up the professional team (health and non-health) of Psychiatry of the Royo Villanova Hospital of Zaragoza in relation to the physiotherapy intervention program, as well as the perceptions they have had about the management of the disease, the barriers and facilitators, including the one proposed in this project, in relation to the participants admitted to their unit. |

| **4.6 Methodology** (**all of** the following fields must be detailed**):**  Study design  Participants: inclusion/exclusion criteria; mode of recruitment (who makes initial contact with participants and how, present study dissemination material, if any), sample size (and justification), randomization (if applicable).  Sources of information: detailed variables (data to be collected), origin of the data, when and how it is collected, to what period of time it refers.  Procedures: detail in a differentiated way the purely assistance procedures from those of the research, present surveys or forms to be used (link in case of online surveys ), risk assessment of the experimental procedures and measures to minimize it.  Statistical analysis  Consideration of the gender perspective: detail the measures adopted so that the results of the study may reflect possible differences by sex/gender.  Limitations of the study  In case of biological samples: detail type and number of samples, how they are collected, where and by whom they are analyzed, when they are destroyed (or final destination). |
| --- |
| ***Study design***  A nested concurrent mixed design study of quantitative dominance will be carried out, following the criteria described in the Mixed Methods of Research (MMARS) guide. The quantitative study will be a quasi-experimental pilot study with a pre-post design. The present study also has an additional qualitative narrative design, in which focus groups will be carried out to analyze the experiences with the intervention, as well as the perceptions about the management of the process, and the barriers and facilitators that the participants have had during the intervention program, and on the other hand, in relation to the perceptions of the professionals who make up the professional team (health and non-health) of Psychiatry of the Royo Villanova Hospital of Zaragoza in relation to the proposed intervention program and how they perceive that it has influenced the management of the disease, the barriers and facilitators, on the patients admitted to their unit.  The study has the authorization of the head of the psychiatry service of Sector I of the Aragonese Health Service and of the Medical Management-Direction of the Royo Villanova University Hospital of Zaragoza (ANNEXES I and II). Likewise, a collaboration agreement has been established between the USJ and the Medical Management-Direction of the Royo Villanova Hospital. A favorable report has been obtained from the ethics committee of the Universidad San Jorge.  NO. 38/3/24-25.  ***Population***  The sample will be composed of consecutive voluntary patients admitted to the Psychiatry Short Stay Unit of the Royo Villanova Hospital (Zaragoza) who meet the following selection criteria:  - Inclusion criteria:   - Over 18 years old. - Admitted to the psychiatric ward of the Royo Villanova Hospital. - Diagnosis of major depressive disorder as a mental illness by a medical professional. - Undergoing regular medical, psychological and pharmacological treatment for his illness. - No need for supervision and control by professional staff of the psychiatric unit during data collection and intervention.   - Exclusion criteria:   - Comorbid presence of physical or mental illness whose clinical characteristics and/or severity impede the understanding and/or follow-up of physical therapy interventions. - Presence of physical or mental dysfunction or disability that prevent or are total or partial contraindications to physical therapy techniques. - Legal incapacity. - Pregnancy.   - Abandonment criteria:   - Express desire of the subject to abandon the study. - Attendance less than 80% of physical therapy intervention sessions. - Injury that prevents the continuation of the sessions. - New disease whose diagnosis and/or severity prevents further study or *exitus*.   For a single-group design, the sample size was calculated on the basis of a moderate effect size for a pilot study (d = 0.5), with a significance level of 0.05 and a power of 0.80. A dropout rate of 25% was also taken into account. Thus, around 40 participants would be needed to detect a significant change, taking into account that they meet the selection criteria where the average length of stay in the short-stay psychiatric unit of the Royo Villanova Hospital is 3 weeks and has a maximum capacity of 25 patients.  The sample (8 people) that will form part of the focus group in the qualitative evaluation of professionals that make up the professional team (health and non-health) of Psychiatry of the Royo Villanova Hospital of Zaragoza will consist of voluntary participants and must meet the following selection criteria:  - Inclusion criteria:   - Healthcare and non-healthcare professionals from the short-stay psychiatry unit of the Royo Villanova Hospital in Zaragoza.   - Exclusion criteria:   - Professionals of the unit who have not been active (single, permanent or temporary employment contract) during the entire physiotherapy intervention.   - Abandonment criteria:   - Express desire of the subject to abandon the study. - Sick leave or *exitus*. - Change of position to another unit or health center.   ***Evaluation***  *Quantitative evaluation*  An initial assessment will be made before the start of the intervention and another at the end of the intervention, always before hospital discharge. The pre-intervention assessment data will be collected on the first days of each admission, for each patient who wishes to participate voluntarily, and the post-intervention assessment data will be collected on the days prior to hospital discharge. This data collection will be carried out over as many weeks as necessary until the sample size is reached. As indicated above, the average hospital stay may be 3 weeks, within which the pre-post evaluation and the program intervention will be done.   - Initial measurements-control variables   Sociodemographic data: age, gender, profession.  Clinical data: height, weight, body mass index, medical diagnosis, pharmacological treatment, other non-pharmacological treatments   - Primary endpoint   QUALITY OF LIFE: **EuroQuol-5D-3L Spanish version (EQ5D-3L Spanish version)**  The EQ-5D-3L health questionnaire is a questionnaire that can be self-administered or carried out through an interview, which allows to know the state of health in relation to several dimensions of the patient (mobility, pain, mental health...). It consists of 5 questions with 3 answer options (good health=1, some problem=2 or health problem=3) where the answers are codified and coefficients are applied according to the answers to reach a reference value; and on the other hand it consists of a vertical visual analogical scale of 20 centimeters, with values from 0=worst state of health to 100=best state of health, where the patient indicates the current value of perceived health. It is a validated tool and widely used in health and research.   - Specific variables   DEPRESSION: **Montgomery-Asberg Depression Rating Scale (MADRS) Spanish validated version.**  This scale is of clinical use for the detection and severity of major depressive disorder. It consists of 10 questions that are asked to the interviewee about cognitive and affective/emotional symptoms with response values between 0 and 6 (0=lowest level of symptomatology and 6=maximum level of symptomatology). Its interpretation will be 0-6 points no depression, 7-19 points mild depression, 20-34 points moderate depression and 35-60 points severe depression.  PAIN: **NRS Scale**  The NRS pain rating scale is a scale that measures the intensity of pain presented by the patient. It is a numerical scale where the patient is asked to rate his pain between 0 and 10. The values reported are classified as: no pain=0; mild pain=1,2; moderate pain=3-5; severe pain=6-8; unbearable pain=9-10.  SELF-EFFICIENCY: **General Self-efficacy scale (General Self-efficacy scale**  The Spanish version of the General Self-efficacy Scale consists of a questionnaire of 10 questions that measure the person's perception of his or her own abilities to manage his or her life in stressful situations. For each question the respondent must answer according to a Likert scale where the answer is scored as 1 totally disagree and 5 totally agree. The score between 27 and 38 points is considered an average of self-efficacy. The higher the score, the higher the perception of self-efficacy.  SATISFACTION: **Health Care Satisfaction Survey for People with Chronic Disease (GCPC-UN-ESU)**  The survey of satisfaction with health care for people with chronic disease (GCPC-UN-ESU) is a validated tool for measuring the level of satisfaction. It contains 19 items with 4 dimensions: care, health education, quality of service provided and level of loyalty to the service. Each item has a score between 1 (not at all satisfied) and 5 (highly satisfied). The higher the score, the higher the satisfaction. It will be measured only in the post-intervention assessment and anonymously.  *Qualitative evaluation*  Different focus groups will be conducted with two different population groups:   - Focus groups only with participating patients with major depressive disorder who have undergone the intervention in the short-stay unit of the Royo Villanova University Hospital in Zaragoza. - Focus groups only with participating professionals from the short-stay psychiatry unit of the Royo Villanova University Hospital in Zaragoza.   Each group of participants will be asked to collaborate in the realization of a focus group. Several focus groups will be conducted with these profiles, each with 7-8 patient participants (depending on the final sample recruited) and 8 professional participants, trying to seek heterogeneity within each group and homogeneity between focus groups. The focus groups will be only of patients or only of professionals.  Focus groups with patients will be conducted sequentially after discharge from the hospital who have undergone the intervention and the pre-post quantitative assessment.  The focus groups with professionals will be carried out within two weeks after the end of the quantitative phase, which will be completed once the required sample size has been reached. The guide of questions for the patient focus groups will deal with the experience with the disease, the perceptions in the management of their process, and the barriers and facilitators they have had during the intervention program (APPENDIX III). The guide of questions for the focus groups of professionals will deal with the patients' perceptions on the management of the disease, and the barriers and facilitators they have been able to observe during the intervention program.  Each focus group will be conducted by one researcher, while another researcher will be in charge of taking field notes and impressions from the participating professionals during the focus group, both of whom will be trained in qualitative research and will have had no previous relationship with the participants. Both researchers will carry out a prior process of researcher positioning or "bracketing", with the objective of ensuring that their previous knowledge and experiences, beliefs and motivation for the research do not influence the data collection and analysis.  The focus group will last approximately 1 hour based on a guide of questions (attached in APPENDIX III) and will be recorded by tape recorder, after the signed consent of each of the participating professionals.  ***Intervention***  All participants will receive the same intervention based on scientific evidence in the short-stay psychiatric unit of the Royo Villanova Hospital in Zaragoza. The intervention will be group-based, with a maximum number of 8 patients per physiotherapist, and will be carried out over a period of 3 weeks, with 2 weekly sessions of approximately 45 minutes. Each session will be divided into two parts:   - Part of therapeutic exercise based on active joint mobility, strength exercises with own weight and/or elastic bands, balance exercises and progressive muscle relaxation exercises. The use of mats, chairs and elastic bands will be necessary. - Part of health education aimed at improving knowledge of their physical condition resulting from mental illness and improving self-management of their physical abilities for better self-efficacy and motor skills.   ***Data analysis***  Statistical analysis of quantitative data will be performed with IBM-SPSS Statistics version 29 software. Data will be expressed as mean and standard deviation or median and interquartile range. The Shapiro-Wilk normality test will be used. Simple and/or repeated measures ANOVA will be conducted to test for differences between pre- and post-test scores, in case the statistical requirements and assumptions of ANOVA (homoscedasticity, etc.) are not met, linear models will be conducted with the post-test score or change score as the outcome variable (DV, outcome) and the pretest scores included as a covariate thus eliminating systematic bias and reducing the error variance. The significance level will be defined as p ≤ 0.05.  Effect size will be calculated using Cohen's d to determine clinical significance: insignificant, small, medium, and large differences will be reflected in effect sizes of <0.2, 0.2-0.5, 0.5-0.8, and >0.8, respectively  For the analysis of the qualitative information obtained in the focus groups, it will be analyzed using the content analysis methodology. The sessions will be transcribed verbatim in their entirety by a single researcher from the recordings made during the focus groups. Initially, one or several complete readings will be made to obtain a global idea of the information recorded and to achieve an immersion in the text. Subsequently, a second reading will be done word by word, in which an inductive coding of the transcripts will be performed, in order to capture the key concepts and thoughts, this process will be carried out by 2 researchers independently. Subsequently, the codes will be grouped by their relationship and linkage into categories. Depending on the relationships between the subcategories, the researchers can compare, combine or organize this larger number of subcategories into a smaller number of categories.  A triangulation of researchers will be carried out, as well as a methodological triangulation (quantitative-qualitative) to improve the quality and reliability of the data. Two authors will perform a contrast and comparison of codes, subcategories and categories, refining and redefining each of them to resolve possible disagreements. Subsequently, the findings will be sent to the rest of the study team for comparison and discussion, in order to reach a consensus among the researchers.  ***Liability***  A specific civil liability insurance for research projects of the Universidad San Jorge covers the participants (Zurich, policy number 86137890).  ***Consideration of the gender perspective***  Equal inclusion between the different sexes will be taken into account during recruitment to obtain a point prevalence by sex similar to the latest available data from the health sector or general population in the city of Zaragoza or the Autonomous Community of Aragon ( )50 . Major depressive disorder is a disease with a higher prevalence in the female sex ( )51 , so it will be taken into account if there are differences according to sex in order to offer specific solutions.  ***Limitations***  The main limitation will be the foreseeable loss of patients who are able to complete the physiotherapy program described, which may lead to lengthening the interventions over time in order to reach the sample size. Another limitation will be the difficulty of controlling the amount of physical exercise performed outside the intervention. The diversity of subtypes of depressive disorder may also influence the results, so the specific diagnosis will be taken into account to minimize this risk. Also the lifestyle prior to admission, whether they have had an active life or not could influence the results. On the other hand, the sample receives standardized treatment for their disease, so that attributing improvements exclusively to therapeutic physical exercise and the health education plan will be difficult to demonstrate.  ***Financial report and source of financing***  The project "Effectiveness of a physiotherapy program based on therapeutic physical exercise and health education aimed at improving quality of life and health status in major depressive disorder: a mixed design study" has a funding of 5700€ (APPENDIX IV) granted through the Internal Call for Projects 2024-2025 of the University San Jorge (APPENDIX V).  The object of financing through which this project was positively evaluated was the dedication of the budget obtained for:   - 2 scientific publications in open access.   This being one of the eligible costs of the projects submitted to this call, and with a deadline of December 31, 2025 for the deposit of results in the entity in charge of publication or registration at the end of the project.  Regarding the use of resources necessary for the implementation of the research project, attached in ANNEX II is the authorization by the head of the Psychiatry Service of Sector I of Zaragoza and the Medical Director of the Royo Villanova University Hospital for the use of the following material within the facilities of the Royo Villanova University Hospital of Zaragoza:   - Use of the audiovisual room of the Psychiatry short-stay service of the Royo Villanova University Hospital of Zaragoza for the assessments, interventions and focus groups of the present study. - Use of the chairs and mats already present in the audiovisual room. - The cost associated with this use would be the cost of air conditioning and lighting of the room during the time required to carry out this study.   They are also included with the respective authorization (ANNEX I) for the collaboration of the staff of the Hospital Universitario Royo Villanova present in this research project. |

| **4.7 Ethical aspects** (risk/benefit balance, **justification in case of requesting exemption from** informed **consent**, care implications, implications for the participant or his/her family, compensation to participants, insurance policy). |
| --- |
| This study respects the fundamental principles set out in the Declaration of Helsinki, the Council of Europe Convention on Human Rights and Biomedicine, the UNESCO Universal Declaration and the law on the protection of personal data of the participants: EU Regulation 679/2016 on the protection of personal data RGPD and Organic Law 3/2018 of 5 December on the Protection of Personal Data and guarantee of digital rights LOPDGDD). The anonymity and confidentiality of all personal data will be maintained.  Participation will be free and voluntary. No participant will receive financial or any other type of compensation for their participation. Each potential participant will be provided with the participant information sheet and, in case of acceptance, the informed consent document, both for patients and professionals.  A person external to the evaluation and the intervention will carry out the process of anonymization of participants. The data protection and informed consent document will be taken into account. Prior to the start of the study, positive assessment of the corresponding ethics committees, as well as all relevant agreements and authorizations, will be available.  All data will be collected in password protected Excel tables located in Microsoft 365 licensed by USJ and anonymized. The PI will create a folder to share with the research team by specific invitation to their @USJ mail. Only the researchers involved will have access. It will be a shared folder with password.  The transcription of the audio recordings will be developed in the fifteen calendar days following the focus groups. Once the transcriptions have been obtained, the recordings will be erased. In addition, recording devices of the Universidad San Jorge will be used for these recordings, under a loan system, and the recordings will be returned without any audio material. The purpose of the recordings is the transcription of the data.  To guarantee the pseudonymization of the participants in the focus groups, the researcher doing the transcriptions will not be present in the focus groups. Each person will be identified with a number and at the beginning of the session will say, for example, "I am voice 1" and so on. Likewise, each person will be asked to say what voice number he or she is before participating in the focus group. This will facilitate transcriptions and anonymity.  For the storage of the personal data of the project, the Microsoft OneDrive service will be used, which is accessed exclusively by the credentials of the researchers of this project. Regular backups will be made in the Microsoft OneDrive space and only the three USJ researchers of the project will have access to the folder containing the database. During the course of the research project, the electronic transmission of personal data or analysis of the project results, which are not strictly necessary for the proper development of the project, will be avoided. |
| **4.8 Schedule and work plan**:   - Stages of development, duration, estimated start and end dates (indicate at least month and year). - Places where the project is planned to be carried out, facilities to be used. |
| \| **Tasks** \| \| --- \| \| Month  1 \| Month 2 \| Month 3 \| Month 4 \| Month 5 \| Month 6 \| Month 7 \| Month 8 \| Month 9 \| Month 10 \| Month 11 \| Month 12 \| \| Acceptance of ethics committee and agreements \| **x** \|  \|  \|  \|  \|  \|  \|  \|  \|  \|  \|  \| \| Recruitment \|  \| **x** \| **x** \| **x** \| **x** \| **x** \| **x** \| **x** \| **x** \| **x** \| **x** \|  \| \| Initial evaluation \|  \| **x** \| **x** \| **x** \| **x** \| **x** \| **x** \| **x** \| **x** \| **x** \| **x** \|  \| \| Intervention \|  \| **x** \| **x** \| **x** \| **x** \| **x** \| **x** \| **x** \| **x** \| **x** \| **x** \|  \| \| Final evaluation \|  \|  \| **x** \| **x** \| **x** \| **x** \| **x** \| **x** \| **x** \| **x** \| **x** \|  \| \| Data analysis \|  \|  \|  \|  \|  \|  \| **x** \| **x** \| **x** \| **x** \| **x** \| **x** \| \| Publications \|  \|  \|  \|  \|  \|  \|  \|  \|  \| **x** \| **x** \| **x** \|   The facilities to be used are the audiovisual room of the psychiatry short-stay service of the Royo Villanova University Hospital.  Estimated project completion in July 2026. |
